# JAK inhibitors and the risk of malignancy: a meta-analysis across disease indications

**DOI:** 10.1136/ard-2023-224049

**Published:** 2023-05-29

**Authors:** Mark D Russell, Christopher Stovin, Edward Alveyn, Olukemi Adeyemi, Chun Kit David Chan, Vishit Patel, Maryam A Adas, Fabiola Atzeni, Kenrick K H Ng, Andrew I Rutherford, Sam Norton, Andrew P Cope, James B Galloway

**Affiliations:** 1 Centre for Rheumatic Diseases, King's College London, London, UK; 2 Rheumatology Unit, University of Messina, Messina, Italy; 3 Department of Medical Oncology, University College London, London, UK

**Keywords:** arthritis, rheumatoid, spondylitis, ankylosing, arthritis, psoriatic, biological therapy, epidemiology

## Abstract

**Objectives:**

To estimate the association of Janus kinase inhibitors (JAKi) with the incidence of malignancy, compared with placebo, tumour necrosis factor (TNF)-α inhibitors (TNFi) and methotrexate.

**Methods:**

Systematic searches of databases were performed, to December 2022, to identify phase II/III/IV randomised clinical trials (RCTs) and long-term extension (LTE) studies of JAKi (tofacitinib, baricitinib, upadacitinib, filgotinib, peficitinib) compared with placebo, TNFi or methotrexate, in adults with rheumatoid arthritis, psoriatic arthritis, psoriasis, axial spondyloarthritis, inflammatory bowel disease or atopic dermatitis. Network and pairwise meta-analyses were performed to estimate incidence rate ratios (IRRs) for malignancy between JAKi and comparators. Bias was assessed using the Cochrane Risk of Bias-2 tool.

**Results:**

In 62 eligible RCTs and 16 LTE studies, there were 82 366 person-years of exposure to JAKi, 2924 to placebo, 7909 to TNFi and 1074 to methotrexate. The overall malignancy incidence rate was 1.15 per 100 person-years in RCTs, and 1.26 per 100 person-years across combined RCT and LTE data. In network meta-analyses, the incidence of all malignancies including non-melanomatous skin cancers (NMSCs) was not significantly different between JAKi and placebo (IRR 0.71; 95% CI 0.44 to 1.15) or between JAKi and methotrexate (IRR 0.77; 95% CI 0.35 to 1.68). Compared with TNFi, however, JAKi were associated with an increased incidence of malignancy (IRR 1.50; 95% CI 1.16 to 1.94). Findings were consistent when analysing NMSC only and when analysing combined RCT/LTE data.

**Conclusions:**

JAKi were associated with a higher incidence of malignancy compared with TNFi but not placebo or methotrexate. Cancers were rare events in all comparisons.

**PROSPERO registration number:**

CRD42022362630.

**WHAT IS ALREADY KNOWN ON THIS TOPIC**

- The ORAL Surveillance trial reported an increased risk of malignancy with tofacitinib compared with tumour necrosis factor (TNF) inhibitors.
- It is unclear whether these results are generalisable to other Janus kinase (JAK) inhibitors and populations, including people aged under 50 years without additional cardiovascular risk factors.

**WHAT THIS STUDY ADDS**

- In this meta-analysis of 78 clinical trials and long-term extension studies of inflammatory joint, skin and bowel diseases, JAK inhibitors did not associate with a higher incidence of malignancy compared with placebo or methotrexate.
- JAK inhibitors were, however, associated with a higher incidence of malignancy compared with TNF inhibitors.
- Cancer events were rare with all treatments, with an overall incidence rate of 1 event per 100 person-years of exposure.

**HOW THIS STUDY MIGHT AFFECT RESEARCH, PRACTICE OR POLICY**

- This study might influence the choice of treatment where the decision is between a JAK inhibitor or TNF inhibitor, particularly in patients at increased risk of malignancy.

## Introduction

Janus kinase inhibitors (JAKi) are therapies that interfere with JAK-STAT signalling by inhibiting one or more JAK enzymes (JAK1, JAK2, JAK3, TYK2). Tofacitinib was the first JAKi approved for the treatment of rheumatoid arthritis (RA), in 2012. Subsequently, four other JAKi have been approved for RA: baricitinib, upadacitinib, filgotinib and peficitinib. Treatment indications have expanded to include psoriatic arthritis (PsA), psoriasis (PsO), axial spondyloarthritis (axSpA), inflammatory bowel disease (IBD) and atopic dermatitis (AD). In head-to-head randomised clinical trials (RCTs) of patients with RA, baricitinib and upadacitinib were better than tumour necrosis factor-α inhibitors (TNFi) for disease activity outcomes.^1 2^

Following licensing of tofacitinib, the Food and Drug Administration mandated a post-marketing surveillance study to evaluate safety, including the risk of malignancy. In the ORAL Surveillance trial, an open-label RCT that compared tofacitinib with TNFi in adults aged over 50 years with RA, non-inferiority criteria were not met for malignancies or major adverse cardiovascular events.^3^ During median follow-up of 4 years, a higher incidence of adjudicated malignancies excluding non-melanomatous skin cancers (NMSCs) was observed with combined tofacitinib doses than with TNFi (4.2% vs 2.9% of patients, respectively; HR 1.48; 95% CI 1.04 to 2.09). Similarly, NMSC incidence was higher with tofacitinib than TNFi (2.2% vs 1.1%, respectively). The most common cancers excluding NMSC were lung cancer with tofacitinib and breast cancer with TNFi.^4^

It remains unclear, however, whether the results of the ORAL Surveillance trial are generalisable to other JAKi, diseases and populations—for example, people aged under 50 years without additional cardiovascular risk factors, in whom the base-rate frequency of malignancy is lower.^4^

This meta-analysis includes RCTs and long-term extension (LTE) studies of all licensed JAKi across treatment indications, to test the hypothesis that JAKi increase the risk of malignancy compared with TNFi, placebo or methotrexate.

## Methods

### Database search strategy

A literature search was conducted using MEDLINE, Embase and Cochrane databases to identify RCTs and LTE studies of JAKi in RA, PsA, PsO, axSpA, IBD (ulcerative colitis and Crohn’s disease) or AD. Search terms are provided within the online supplemental appendix. The search was limited to articles published from database inception to 9 December 2022. Additional trials were searched for in study references and trial databases. The search was performed in accordance with the Preferred Reporting Items for Systematic Reviews and Meta-Analyses,^5^ and registered with the International Prospective Register of Systematic Reviews (PROSPERO registration ID: CRD42022362630).

10.1136/ard-2023-224049.supp1 Supplementary data

### Eligibility criteria and study selection

Eligible studies were phase II, III or IV RCTs or LTE studies of JAKi (tofacitinib 5 mg or 10 mg two times per day or 11 mg once daily; baricitinib 2 mg or 4 mg once daily; upadacitinib 15 mg or 30 mg once daily; filgotinib 100 mg or 200 mg once daily; peficitinib 100 mg or 150 mg once daily) studied in adults with RA, PsA, PsO, axSpA, IBD or AD that included comparisons with placebo, methotrexate monotherapy or TNFi. For LTE studies where only interim data were published, or where the same dataset was reported more than once, the most recently published data were included. Studies not reporting malignancy outcomes were not eligible. Case–control studies, observational cohort and registry studies were excluded.

Records were managed in Rayyan (Cambridge, USA). Study titles and abstracts were screened independently by two investigators (CS, OA). The full texts of relevant studies were retrieved and assessed for eligibility. Disagreements were resolved through involvement of a third reviewer (MDR).

### Data collection

Data were extracted independently, in duplicate, by three investigators (CS, MDR, EA), with involvement of a fourth reviewer (JG) to resolve discrepancies when required. Data were identified from publications, with cross-referencing to published information in ClinicalTrials.gov. Data extracted included: study characteristics; demographics; intervention and comparator treatments; person-years of exposure and malignancy events (benign tumours and dysplasias without malignancy, where specified, were excluded; in situ malignancies were included). For studies in which person-years of exposure were not reported, exposures were estimated from the per-protocol participant disposition (ie, according to the received treatment), whereby the number of participants completing each study period was multiplied by the duration of exposure. This approach was chosen over estimations based on intention-to-treat disposition, as the objective was to assess medication safety.

Risk of bias was assessed using the Cochrane Risk of Bias-2 tool.^6^ This was performed independently for eligible RCTs by two reviewers (MA, VP), with disagreements resolved by involvement of a third reviewer (JG).

### Outcomes

The primary outcome was the incidence of all malignancies including NMSC. Secondary outcomes were all malignancies excluding NMSC, NMSC only and haematological malignancies (including lymphoma) only.

### Statistical analysis

Meta-analysis was performed to estimate the risk of malignancy between JAKi and comparators. Incidence rate ratios (IRRs) for malignancies were reported separately for pooled phase II/III/IV RCT data (ie, not including LTE data) and for combined phase II/III/IV RCT and LTE data. In LTE studies without long-term comparator data (14 of 16 studies), comparator data from the original RCTs were included for comparison. Meta-analysis was also performed to estimate the effect of individual JAKi medications on the risk of malignancies, relative to placebo.

Network meta-analysis was conducted using restricted maximum likelihood models to compare outcomes across studies, combining direct and indirect comparisons between treatments. Between-treatment IRRs for malignancies (with 95% CIs) were described graphically using forest plots. The number of studies for each treatment and comparison was described in network plots. A fixed continuity correction of 0.1 was applied to each arm of studies that had one or more groups with zero events. Each drug was ranked based on estimated probabilities using the parameters derived from the network meta‐analysis; these were summarised by calculating the surface under the cumulative ranking curve (SUCRA). Network meta-analysis consistency assumptions were tested at the overall level (Wald test for inconsistency) and for each treatment comparison (node-splitting model).

Pairwise meta-analysis was conducted to estimate IRRs for direct treatment comparisons only. Pooled effect estimates were calculated from random-effects DerSimonian and Laird models with fixed continuity corrections of 0.1 for studies with zero events. Alternative models were explored, including models with treatment-arm continuity corrections for zero events and conditional generalised linear mixed-effects logistic regression models with exact likelihood; however, these made no meaningful difference to estimates. Heterogeneity was reported using I² statistics. Funnel plots and Egger’s test for funnel asymmetry were performed to explore publication bias.

Additional sensitivity meta-analyses were performed, as follows: (1) ‘leave-one-out’ meta-analysis to investigate the influence of individual studies on pooled estimates^7^; (2) analysis of studies conducted in participants with RA only (ie, the largest, single treatment indication); (3) exclusion of tofacitinib 10 mg two times per day and upadacitinib 30 mg once daily doses, which are not approved for some treatment indications; and (4) exclusion of malignancy events that occurred within 6 months of treatment initiation, to account for a potential induction period between receiving treatment and developing cancer. Random-effects meta-regression was performed to explore the impact of differences in age and sex between intervention and comparator groups of each RCT on the relative incidence of malignancy.

All statistical analyses were conducted using Stata V.17 (StataCorp, USA).

### Patient and public involvement

Patients and the public were not involved in the design, conduct or reporting of this research, but are being involved in the dissemination of its findings.

## Results

### Study characteristics

Sixty-two RCTs were included, in addition to 16 LTE studies: tofacitinib (RCT n=19; LTE n=4); baricitinib (RCT n=12; LTE n=2); upadacitinib (RCT n=16; LTE n=6); filgotinib (RCT n=9; LTE n=3) and peficitinib (RCT n=6; LTE n=1). Details of included studies and a flow chart of study selection are shown in online supplemental table 1 and online supplemental figure 1, respectively. Of 62 eligible RCTs, 56 had placebo groups, 10 had TNFi groups (adalimumab n=8; etanercept n=1; adalimumab or etanercept n=1) and 5 had methotrexate monotherapy groups (de-novo methotrexate n=4; maintenance methotrexate n=1).

For combined RCT and LTE data, there were 82 366 person-years of exposure to JAKi groups (mean: 1056 person-years per study; n=36 681 participants; mean follow-up: 118 weeks; SD for follow-up: 67 weeks): tofacitinib 41 585 person-years (n=14 225); baricitinib 16 992 person-years (n=6301); upadacitinib 11 533 person-years (n=9810); filgotinib 9976 person-years (n=5166); peficitinib 2280 person-years (n=1179). There were 2924 person-years of exposure to placebo (mean: 52 person-years per study; n=8752 participants; mean follow-up: 20 weeks; SD 11 weeks), 7909 person-years for TNFi (mean: 791 person-years; n=3811; mean follow-up: 111 weeks; SD 80 weeks), and 1074 person-years for methotrexate (mean: 215 person-years; n=1342; mean follow-up: 52 weeks; SD 28 weeks). Of 62 eligible RCTs, 39 (62.9%) were deemed at low risk of bias across all domains, 16 (25.8%) were considered to have some bias and 7 (11.3%) had at least one domain with high risk of bias (online supplemental table 2).

Across all study groups of eligible RCTs, there were 497 malignancy events, corresponding to an incidence rate (IR) of 1.15 cancers per 100 person-years of exposure. There were 1189 malignancy events across combined RCT and LTE data (IR: 1.26 cancers per 100 person-years).

### Network meta-analysis

**Figure 1:**
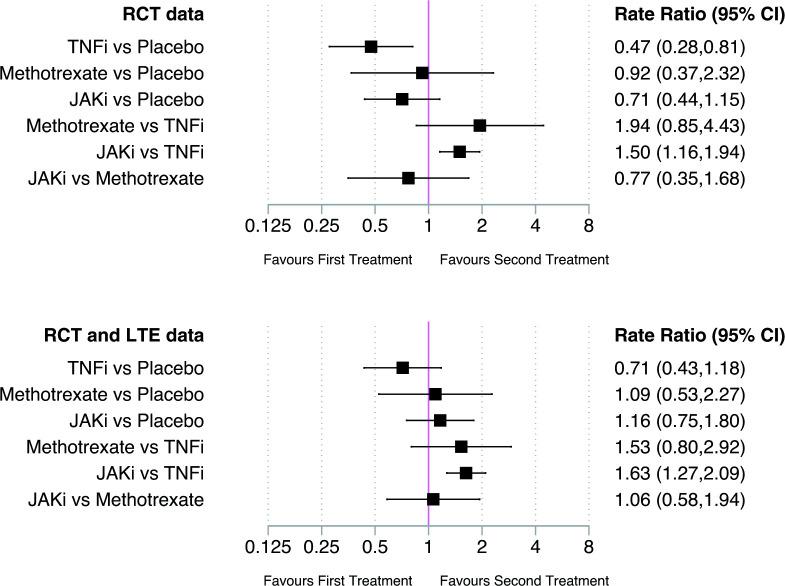
Network meta-analysis estimates of the risk of all malignancies including non-melanomatous skin cancers between study treatments in eligible RCTs (top panel) and combined RCT and LTE studies (bottom panel); expressed as incidence rate ratios with 95% CIs and depicted graphically as a forest plot. JAKi, Janus kinase inhibitor; LTE, long-term extension; RCT, randomised clinical trial; TNFi, tumour necrosis factor-α inhibitor.

**Figure 2:**
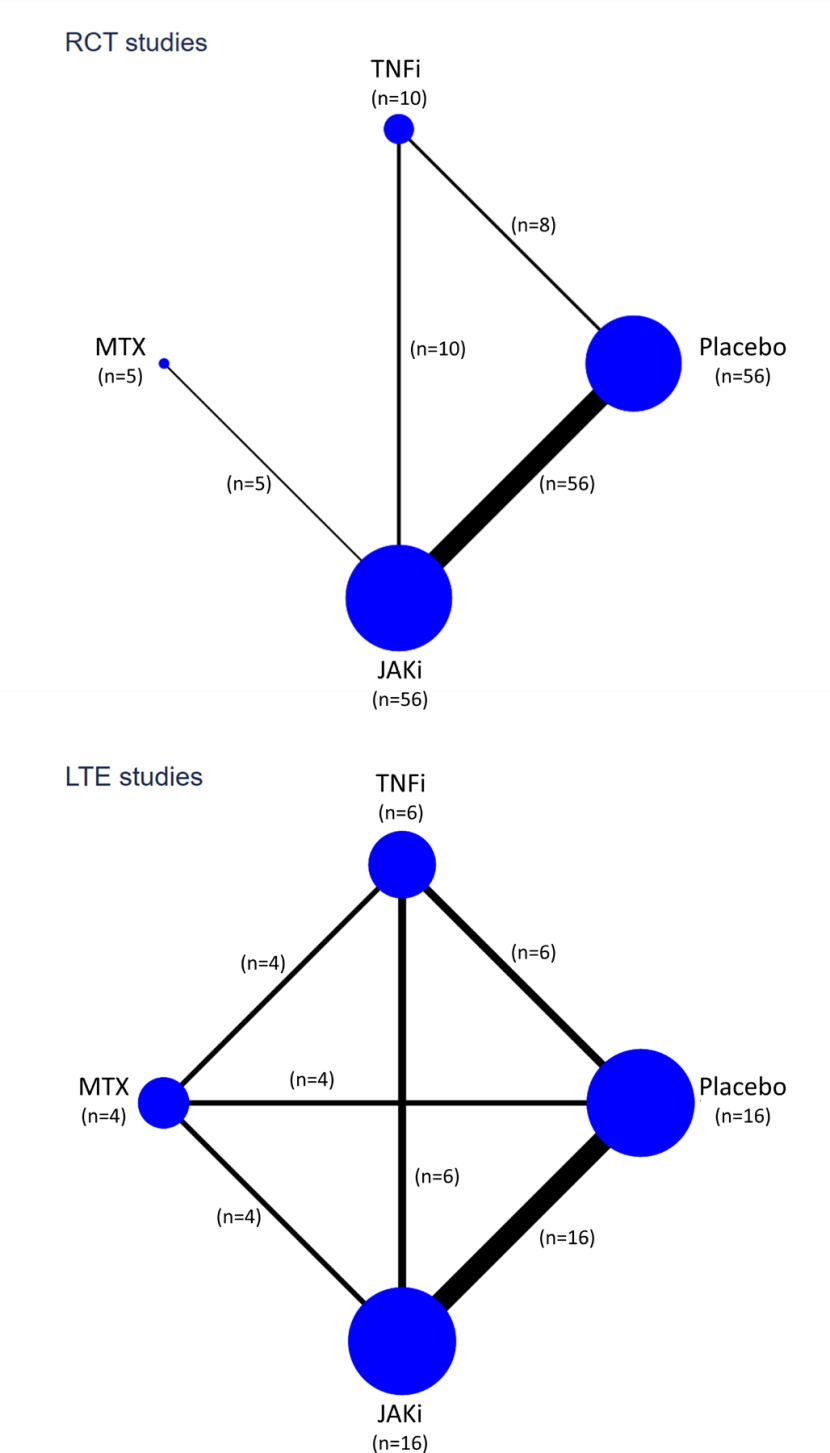
Network plots, depicting the number of studies for each treatment (node size) and number of treatment comparisons (edge thickness) in eligible RCT studies (top panel) and LTE studies (bottom panel). In LTE studies without long-term comparator data, comparator data from the original RCTs were included. JAKi, Janus kinase inhibitor; LTE, long-term extension; MTX, methotrexate; RCT, randomised clinical trial; TNFi, tumour necrosis factor-α inhibitor.

Estimates of malignancy risk from network meta-analyses that compared: (1) all eligible RCTs and (2) combined RCT/LTE data, are shown in figure 1. Using the SUCRA approach to rank malignancy risk between treatments, TNFi associated with the lowest risk of malignancy, followed by JAKi, methotrexate, then placebo in network meta-analyses of eligible RCTs (online supplemental table 3). For combined RCT/LTE data, malignancy risk was lowest with TNFi, followed by placebo, methotrexate, then JAKi (online supplemental table 3). Network plots depicting treatment comparisons are shown in figure 2. Inconsistency was absent in both global and local tests of network meta-analysis assumptions.

### Malignancy risk comparing JAKi with placebo

**Figure 3:**
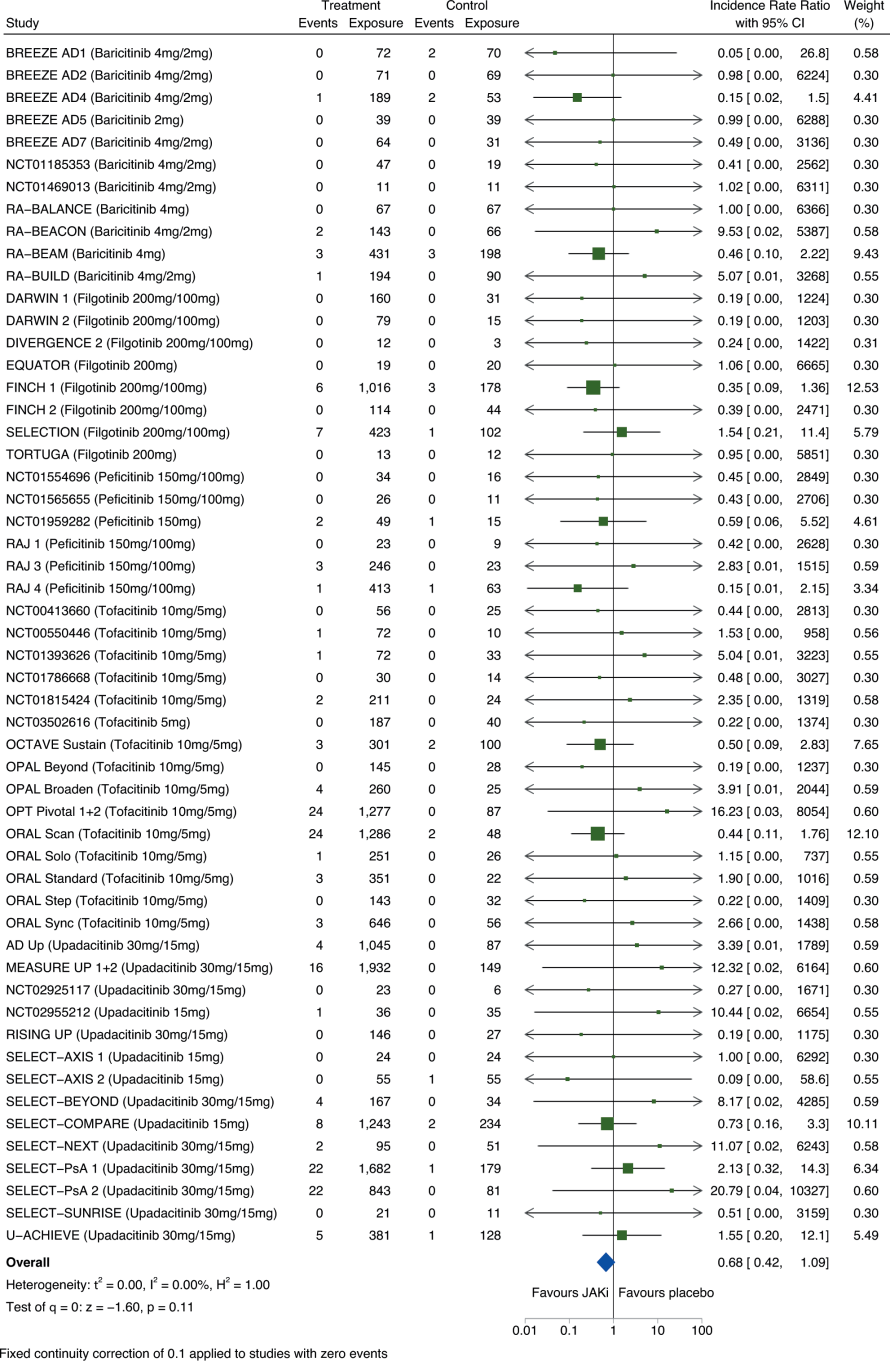
Pairwise meta-analysis of the risk of all malignancies including non-melanomatous skin cancers between JAKi and placebo groups of eligible RCTs; expressed as incidence rate ratios with 95% CIs and depicted graphically as a forest plot. Exposure is reported in person-years. The relative weighting of each study from a random-effects model is shown. A fixed continuity correction of 0.1 was used for studies with zero events. Heterogeneity between studies was assessed using I^2^ statistics. Further details and references for included studies are provided within online supplemental table 1. JAKi, Janus kinase inhibitor; RCTs, randomised clinical trials.

In network meta-analysis of eligible RCTs, there was no significant difference in the risk of all malignancies including NMSC between JAKi and placebo (IRR 0.71; 95% CI 0.44 to 1.15) (figure 1). Similar estimates were obtained from pairwise meta-analysis of direct JAKi–placebo comparisons (n=56) (figure 3). Study heterogeneity was low (I^2^=0%).

Comparable findings were observed from network meta-analyses of all malignancies excluding NMSC (IRR 0.74; 95% CI 0.41 to 1.35; online supplemental figure 2) and for NMSC only (IRR 0.61; 95% CI 0.29 to 1.28; online supplemental figure 3). Haematological malignancies, including lymphomas, were not meta-analysed separately due to low event frequency in JAKi and placebo groups (IR 0.06 vs 0.04 per 100 person-years, respectively).

Similarly, in network meta-analyses of combined RCT/LTE data, there were no significant differences in malignancy risk between JAKi and placebo for: (1) all malignancies including NMSC (IRR 1.16; 95% CI 0.75 to 1.80) (figure 1 and online supplemental figure 4 for pairwise comparisons); (2) all malignancies excluding NMSC (IRR 0.97; 95% CI 0.57 to 1.66; online supplemental figure 2) or (3) NMSC only (IRR 1.00; 95% CI 0.51 to 1.96; online supplemental figure 3).

**Figure 4:**
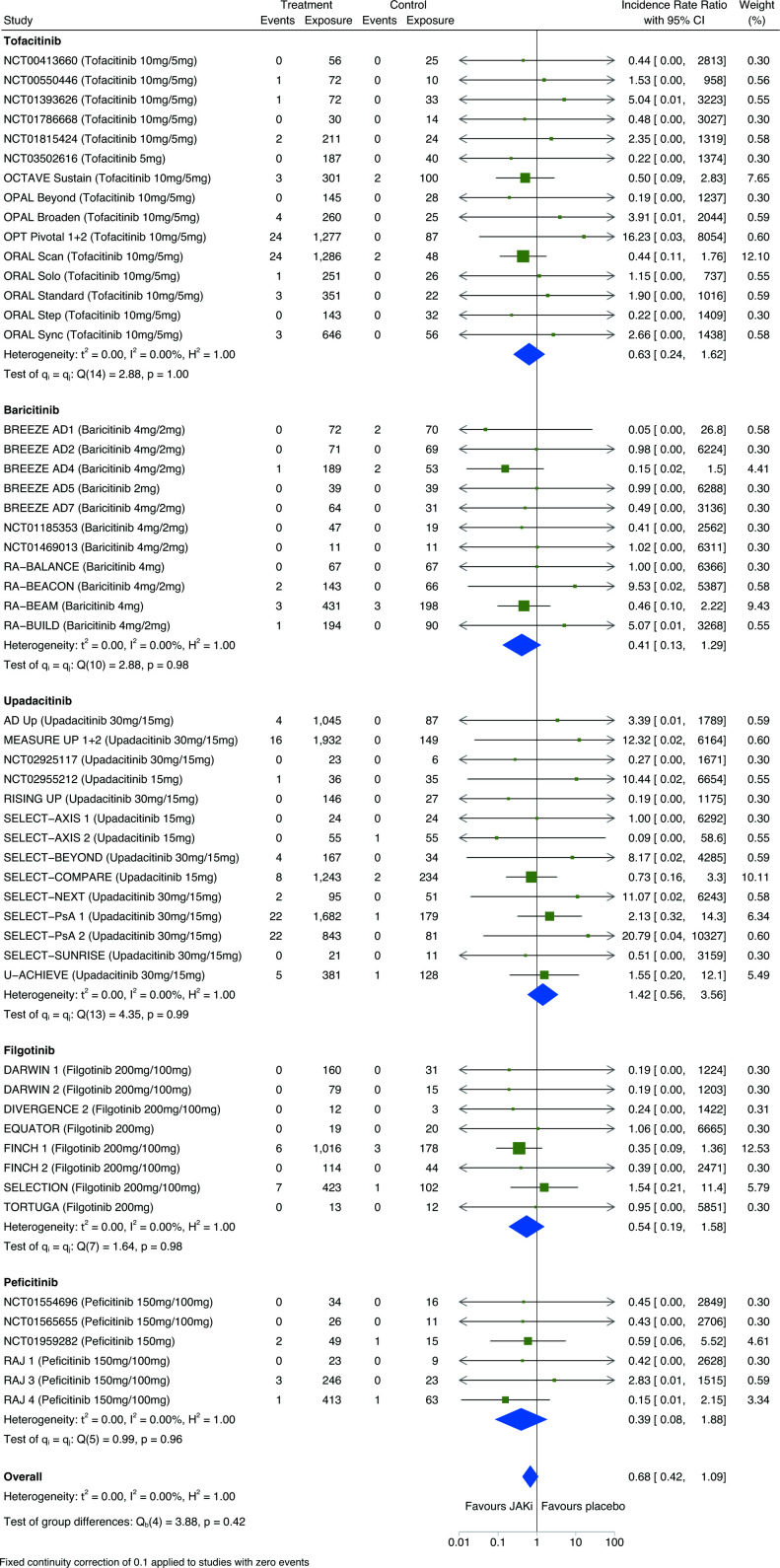
Pairwise meta-analysis of the risk of all malignancies including non-melanomatous skin cancers between individual JAKi medications and placebo groups of eligible RCTs; expressed as incidence rate ratios with 95% CIs and depicted graphically as a forest plot. Exposure is reported in person-years. The relative weighting of each study from a random-effects model is shown. A fixed continuity correction of 0.1 was used for studies with zero events. Heterogeneity between studies was assessed using I^2^ statistics. Further details and references for included studies are provided within online supplemental table 1. JAKi, Janus kinase inhibitor; RCTs, randomised clinical trials.

Pairwise meta-analysis of individual JAKi medications (tofacitinib, baricitinib, upadacitinib, filgotinib, peficitinib) showed no significant differences in malignancy incidence, compared with placebo, in eligible RCTs (figure 4) or combined RCT/LTE data (online supplemental figure 5); however, there was considerable uncertainty in estimates.

Sensitivity analyses that excluded individual studies in turn made no substantial differences to estimates (online supplemental figures 6 and 7). Similarly, no significant differences in malignancy risk between JAKi and placebo were observed in sensitivity analyses that: (1) excluded cancers within the first 6 months of treatment (online supplemental figure 8); (2) included only participants with RA (online supplemental figure 9); or (3) excluded tofacitinib 10 mg two times per day and upadacitinib 30 mg once daily doses (online supplemental figure 10). Funnel plots were not suggestive of significant publication bias or small study effects (online supplemental figure 11; Egger’s test of funnel asymmetry: p=0.95 for RCTs; p=0.15 for RCT/LTE; of note, however, malignancy events were not the primary outcome for the purpose of registration in the vast majority of studies).

### Malignancy risk comparing JAKi with TNFi

**Figure 5:**
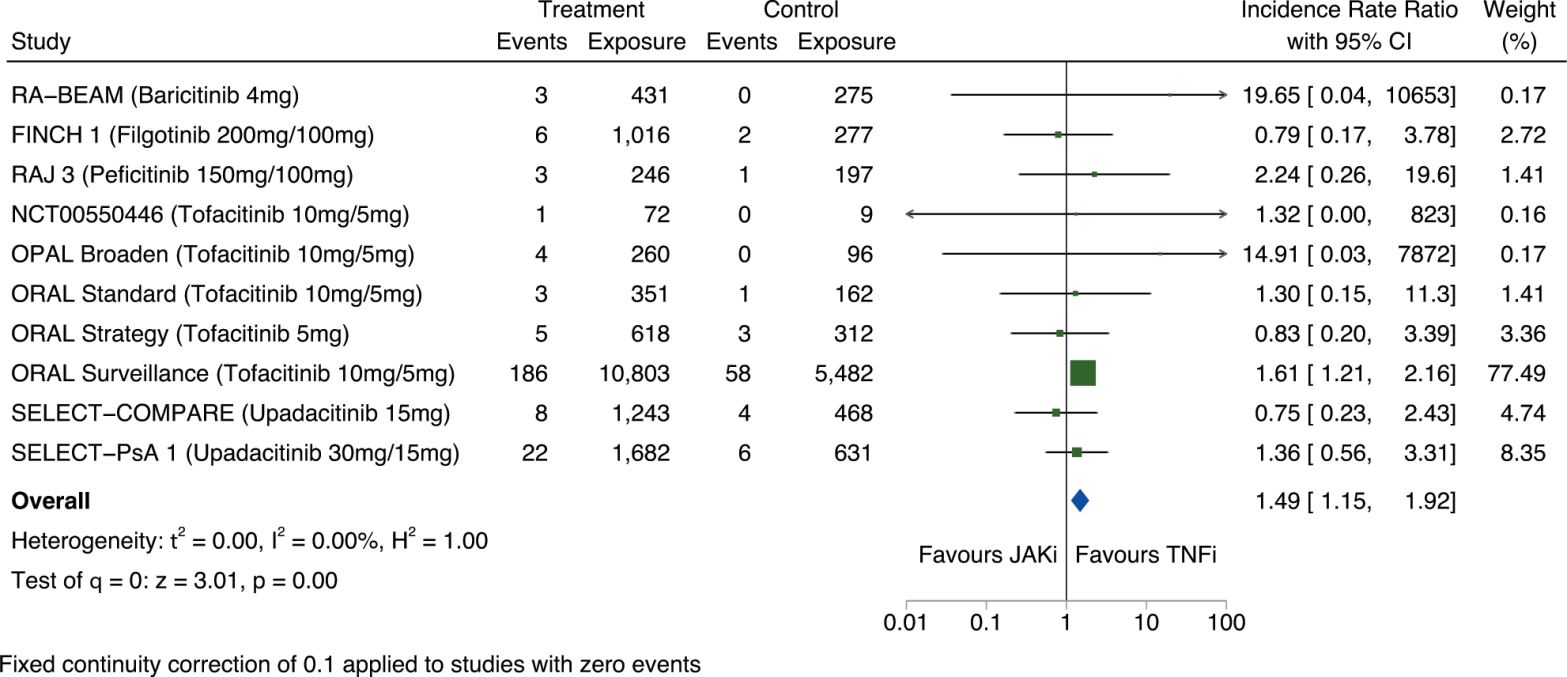
Pairwise meta-analysis of the risk of all malignancies including non-melanomatous skin cancers between JAKi and TNFi groups of eligible RCTs; expressed as incidence rate ratios with 95% CIs and depicted graphically as a forest plot. Exposure is reported in person-years. The relative weighting of each study from a random-effects model is shown. A fixed continuity correction of 0.1 was used for studies with zero events. Heterogeneity between studies was assessed using I^2^ statistics. Further details and references for included studies are provided within online supplemental table 1. JAKi, Janus kinase inhibitor; RCTs, randomised clinical trials; TNFi, tumour necrosis factor-α inhibitor.

In network meta-analyses of RCTs, JAKi associated with a significantly higher incidence of all malignancies including NMSC compared with TNFi (IRR 1.50; 95% CI 1.16 to 1.94) (figure 1). Similar estimates were obtained from pairwise meta-analyses of direct JAKi–TNFi comparisons (n=10; figure 5).

The incidence of malignancies excluding NMSC was higher with JAKi than TNFi, but with a CI that crossed 1 (IRR 1.34; 95% CI 0.99 to 1.82; online supplemental figure 2). NMSC incidence was significantly higher with JAKi than TNFi (IRR 1.93; 95% CI 1.19 to 3.12; online supplemental figure 3). Haematological malignancies were not meta-analysed separately due to low event frequency in JAKi and TNFi groups (IR 0.03 vs 0.06 events per 100 person-years, respectively).

When meta-analysing combined RCT/LTE data, the incidence of malignancy was higher with JAKi than TNFi for: (1) all malignancies (IRR 1.63; 95% CI 1.27 to 2.09; figure 1 and online supplemental figure 12); (2) all malignancies excluding NMSC (IRR 1.43; 95% CI 1.06 to 1.92; online supplemental figure 2); and (3) NMSC only (IRR 2.12; 95% CI 1.32 to 3.41; online supplemental figure 3).

Sensitivity analyses that excluded individual studies demonstrated the large influence of one study: ORAL Surveillance^3^ (online supplemental figure 13). This study carried a weight of 77.5% and 72.9% in pairwise meta-analyses of RCT data and combined RCT/LTE data, respectively. When excluding this study, estimates remained in the same direction of effect but were no longer statistically significant: RCT data (IRR 1.11; 95% CI 0.65 to 1.92); RCT/LTE data (IRR 1.60; 95% CI 0.99 to 2.58). The incidence of malignancy remained significantly higher with JAKi than TNFi in sensitivity analyses that: (1) excluded cancers occurring within the first 6 months (online supplemental figure 8); (2) included only participants with RA (online supplemental figure 9); and (3) excluded tofacitinib 10 mg two times per day and upadacitinib 30 mg once daily doses (online supplemental figure 10).

### Malignancy risk comparing JAKi with methotrexate

**Figure 6:**
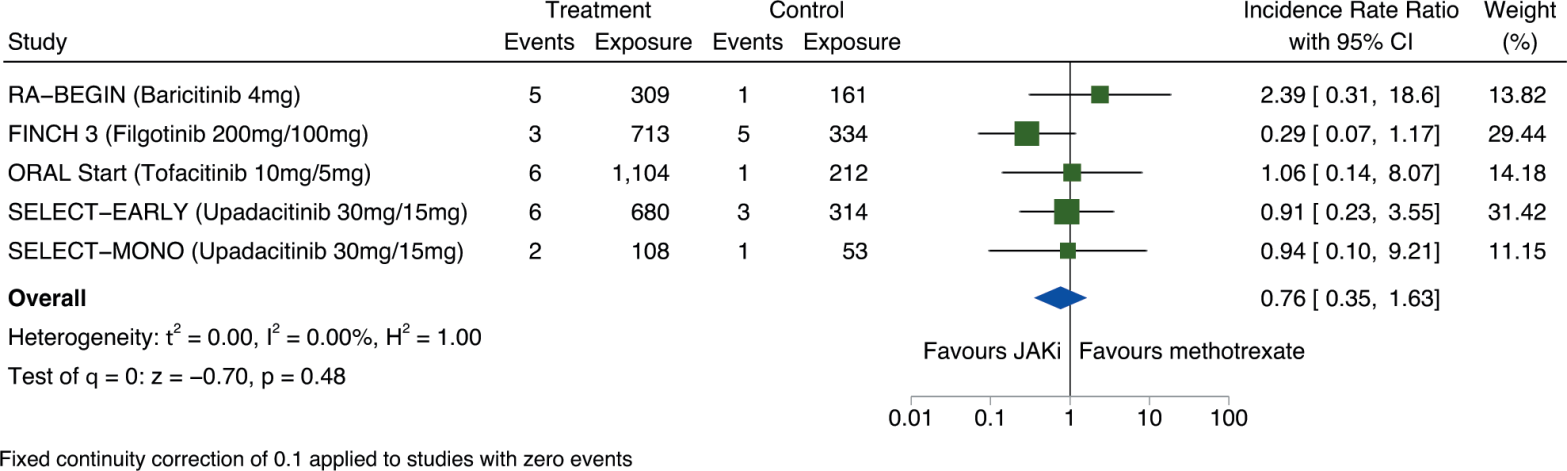
Pairwise meta-analysis of the risk of all malignancies including non-melanomatous skin cancers between JAKi and methotrexate groups of eligible RCTs; expressed as incidence rate ratios with 95% CIs and depicted graphically as a forest plot. Exposure is reported in person-years. The relative weighting of each study from a random-effects model is shown. A fixed continuity correction of 0.1 was used for studies with zero events. Heterogeneity between studies was assessed using I^2^ statistics. Further details and references for included studies are provided within online supplemental table 1. JAKi, Janus kinase inhibitor; RCTs, randomised clinical trials.

When comparing JAKi with methotrexate in network meta-analyses of RCT data, there was no significant difference in the risk of all malignancies including NMSC (IRR 0.77; 95% CI 0.35 to 1.68) (figures 1 and 6). Similarly, with combined RCT/LTE data, there were no differences in malignancy risk between JAKi and methotrexate (IRR 1.06; 95% CI 0.58 to 1.94) (figure 1 and online supplemental figure 14). The small number of studies resulted in wide CIs for analyses of malignancies excluding NMSC (online supplemental figure 2) and for NMSC only (online supplemental figure 3).

### Other comparisons

Estimates from network meta-analyses for other treatment comparisons (TNFi vs placebo; TNFi vs methotrexate; methotrexate vs placebo) are shown in figure 1. Eight RCTs included comparisons of TNFi and placebo, which contributed to 13.2% of the effect estimate in network meta-analyses. In network meta-analyses of RCT data, TNFi associated with a lower incidence of all malignancies compared with placebo (IRR 0.47; 95% CI 0.28 to 0.81). In analyses of RCT/LTE data, the incidence of malignancy with TNFi was numerically lower than placebo, but not significantly so (IRR 0.71; 95% CI 0.43 to 1.18).

Meta-regression was performed to evaluate for evidence of effect modification between age, sex and treatment on IRRs for malignancy. No statistically significant associations were found for these characteristics in JAKi versus placebo, JAKi versus TNFi or JAKi versus methotrexate comparisons (online supplemental table 4).

## Discussion

In this meta-analysis, the risk of malignancy did not differ significantly between JAKi and placebo or between JAKi and methotrexate in RCT or LTE studies. Compared with TNFi, however, JAKi associated with a higher incidence of malignancy. This observation was primarily due to the ORAL Surveillance trial, which compared tofacitinib with TNFi in people with RA aged over 50 years who had additional cardiovascular risk factors. When excluding this study, effect estimates remained in the direction of higher malignancy incidence with JAKi compared with TNFi, although this difference was no longer statistically significant.

ORAL Surveillance was the only study in our analyses to show a statistically significant increase in the incidence of malignancy with JAKi, relative to its comparator (TNFi). Of 10 studies that directly compared JAKi with TNFi, 7 were in the direction of harm for JAKi; however, CIs for all studies were wide, reflecting in part the rarity of cancer events. The large sample size of ORAL Surveillance, enriched for a population at risk of the event of interest, will have increased power to detect small differences in rare events. To explore this, we performed sensitivity analyses using a ‘leave-one-out’ approach, which systematically removes one study at a time and presents the summary effect estimates with that study excluded. It is essential that interpretation of our primary analysis finding (ie, increased malignancy incidence with JAKi vs TNFi) is considered in the context of one particularly dominant study, which had a different objective and study population to other included studies.

One possible explanation for a finding of higher malignancy incidence with JAKi than TNFi could be that JAKi are harmful and predispose to malignancy. Conversely, the association of JAKi on malignancy could be neutral or even protective, although relatively less so than TNFi. In favour of a neutral effect of JAKi on cancer risk were our findings of no significant differences in malignancy risk between JAKi and placebo or between JAKi and methotrexate. Additionally, our network meta-analyses suggested a lower incidence of malignancy with TNFi than placebo.

There are likely to be many, as yet not fully understood, pathways that influence the risk of malignancy with JAK inhibition. This, in turn, could vary between individual JAKi targeting different JAK-STAT pathways. In vitro, tofacitinib abrogates natural killer (NK) cell maturation and tumour lysis capacity, which play important roles in anti-cancer immunosurveillance.^8^ Inhibition of NK cell proliferation varies depending on JAK selectivity: in cellular assays, tofacitinib showed the largest dose-dependent inhibition of NK cell proliferation, followed by upadacitinib, baricitinib and filgotinib.^9^ In mouse models, continuous administration of tofacitinib increased metastatic lung surface nodules, whereas etanercept—a TNFi—did not.^10^ In reality, the complexity of JAK-STAT signalling affecting multiple overlapping pathways^11^ means that we are far from understanding the important question of intraclass JAKi differences in malignancy risk.

Mechanistically, TNF-α has actions that could promote or inhibit cancer development.^12 13^ In 2006, concerns were raised when a meta-analysis of RCT data for two TNFi (infliximab and adalimumab) reported a threefold increased odds of malignancy compared with placebo in RA.^14^ Several early observational studies reported numerically increased incidences of NMSC and lymphoproliferative cancers with TNFi, compared with other treatments (eg, methotrexate), although with wide CIs.^15^ Many subsequent studies with larger person-years of exposure and adjustment for confounders have reported no increased incidence of solid organ or lymphoproliferative cancers with TNFi.^16 17^ Additionally, a small number of claims-based studies from South Korea and Taiwan have reported lower cancer incidence with TNFi.^18–20^ Taken together, these findings highlight the methodological challenges of separating cancer risk associated with medications from that of the underlying disease,^21–23^ disease activity^12^ and shared risk factors (eg, smoking).^24^ Multiple pathways likely contribute to cancer risk in people on immunosuppressive medications, and risks may vary according to cancer type. For example, some treatments may lower the risk of certain cancers (eg, lymphoma) through improved disease control,^25^ while increasing the risk of other cancers linked to immunosuppression (eg, NMSC). Additionally, differences in disease phenotype and prior treatment exposure between early placebo-controlled RCTs of TNFi and recent RCTs of JAKi make comparisons challenging.

Previous integrated safety analyses have reported malignancy event rates for individual JAKi in the treatment of RA.^26–29^ In an analysis of the tofacitinib clinical trial programme in RA, malignancy incidence rates remained stable over follow-up of up to 9.6 years (median exposure, 3.1 years), while comparable malignancy rates were observed for tofacitinib 5 mg and 10 mg two times per day doses.^26^ Similarly, malignancy incidence rates remained consistent during long-term follow-up in integrated analyses of baricitinib (median exposure, 4.6 years; maximum exposure, 9.3 years) and filgotinib (median exposure, 1.6 years; maximum exposure, 5.6 years).^27 28^ Phase III RA trial data for upadacitinib (mean exposure, 1 year; maximum exposure, 2.5 years) reported comparable malignancy rates between upadacitinib 15 mg daily, placebo, methotrexate and adalimumab; however, numerically higher cancer rates, including NMSC, were observed with upadacitinib 30 mg daily, relative to 15 mg daily dosing.^29^

For rare events with long latency periods, such as cancer, longitudinal real-world data are important, but they remain limited currently. In the US CorEvitas RA registry, data from 2012 to 2019 showed comparable malignancy rates between patients with RA initiating tofacitinib versus TNFi or other biological drugs (4505 vs 16 671 person-years of exposure, respectively).^30^ In the STAR-RA Study of US insurance claims data spanning 2012–2020, an HR of 1.01 (95% CI 0.83 to 1.22) was reported for incident malignancies associated with tofacitinib versus TNFi in RA.^31^ Recent data from the ARTIS registry in Sweden reported HRs of 0.94 (95% CI 0.65 to 1.38) for malignancies excluding NMSC, and 1.39 (95% CI 1.01 to 1.91) for incident NMSC, when comparing JAKi (predominantly baricitinib) with TNFi in participants with RA (4022 vs 21 389 person-years of exposure, respectively).^32^

Our study had several strengths. We combined data on multiple licensed JAKi without restriction by disease indication. Data on TNFi and methotrexate groups were included, facilitating comparisons between medications with distinct mechanisms of action. RCT and LTE data were incorporated, maximising patient-years of exposure and increasing the power to detect differences in rare events. While the ORAL Surveillance trial was influential in comparisons of JAKi and TNFi groups, this study represented only 13% of overall person-time exposure and 17% of cancer events for combined JAKi groups. We also used meta-regression to explore differential impacts of age or sex on cancer risk with JAKi (ie, over and above that of TNFi, placebo or methotrexate), but found no association to suggest this.

This study has several limitations. First, while there were over 80 000 person-years of exposure to JAKi, there were only 2900 person-years of exposure to placebo, reflecting the relatively short duration of placebo exposure in most RCTs. Second, cancers often take several years to develop. Participants entering clinical trials are screened upon entry, and those with symptoms suggestive of malignancy are unlikely to be included. As such, one might expect to observe a lower rate of malignancy in earlier stages of clinical trials, with rates increasing over time. This could favour study arms with relatively short duration of follow-up (eg, placebo), and potentially bias towards seeing an observed difference when no true difference exists. Of note, we did not observe a significantly increased incidence of malignancy with JAKi versus placebo in either RCT or combined RCT/LTE data, suggesting this potential bias is unlikely to have substantially altered the clinical interpretation of our findings. Third, despite being the largest meta-analysis of JAKi and malignancy risk to date, CIs were wide in several analyses, particularly for individual JAKi medications, with estimates spanning both harmful and protective effects. While data from a large post-marketing surveillance study were available for tofacitinib (ORAL Surveillance), similar data were not available for other JAKi at the time of these analyses. Caution is therefore needed when interpreting comparisons between individual JAKi medications. Fourth, for studies that did not report person-years of exposure by group, exposure was estimated according to assigned treatment. This approach was selected because the objective of this study was to assess medication safety; however, the potential for selection bias with this approach must also be considered (for example, conditioning on post-randomisation exposures). Fifth, the relatively small number of events by cancer type and heterogeneous reporting between studies precluded more detailed analyses of the risk of cancer subtypes.

## Conclusion

JAKi were associated with a higher incidence of malignancy compared with TNFi, but not compared with placebo or methotrexate. This association was driven primarily by the results of one large study, ORAL Surveillance. Importantly, malignancies were rare events across all treatment groups.

## Data Availability

Data are available upon reasonable request. All data used in this study are available online within the provided reference list, and can be shared upon reasonable request.
