# Supplementary material for: JAK inhibitors and the risk of malignancy: a meta-analysis across disease indications
Source: Ann Rheum Dis. 2023 May 29;82(8):1059–67. doi: 10.1136/ard-2023-224049 (PMC10359573; doi:10.1136/ard-2023-224049)
Supplement: Supplementary data [file ard-2023-224049supp001.pdf]

Supplementary Table S1. Details of included eligible studies identified from the systematic literature search.

Author (study year/name/reference)	Study location	Study phase	Study duration, weeks	Disease	Doses included in analyses, mg	Comparators	Number participants	Mean age, years	Female participants, %
**Tofacitinib**									
Kremer (2013) (ORAL Sync)[1]	Worldwide	3	52	RA	5mg/10mg	PBO	795	52	83
Gladman (2017) (OPAL Beyond)[2]	Worldwide	3	26	PsA	5mg/10mg	PBO	394	50	53
Mease (2017) (OPAL Broaden)[3]	Worldwide	3	52	PsA	5mg/10mg	PBO, ADA	422	47	56
Sandborn (2017) (OCTAVE Sustain)[4]	Worldwide	3	52	UC	5mg/10mg	PBO	593	43	46
Deodhar (2021) (NCT03502616)[5]	Worldwide	3	48	AxSpA	5mg	PBO	269	42	13
Fleischmann (2012) (ORAL Solo)[6]	Worldwide	3	26	RA	5mg/10mg	PBO	610	52	87
van Vollenhoven (2012) (ORAL Standard)[7]	Worldwide	3	52	RA	5mg/10mg	PBO, ADA	717	53	84
Lee (2014) (ORAL Start)[8]	Worldwide	3	104	RA	5mg/10mg	MTX	956	50	80
Fleischmann (2017) (ORAL Strategy)[9]	Worldwide	3b/4	56	RA	5mg	ADA	1146	50	83
Papp (2015) (OPT Pivotal 1+2)[10]	Worldwide	3	52	PsO	5mg/10mg	PBO	1859	46	30
Panés (2017) (NCT01393626)[11]	Worldwide	2b	38	Crohn's	5mg/10mg	PBO	279	40	46
Burmester (2013) (ORAL Step)[12]	Worldwide	3	26	RA	5mg/10mg	PBO	399	55	86
Zhang (2017) (NCT01815424)[13]	Asia	3	52	PsO	5mg/10mg	PBO	266	41	26
van der Heijde (2019) (ORAL Scan)[14]	Worldwide	3	104	RA	5mg/10mg	PBO	797	53	85
Ytterberg (2022) (ORAL Surveillance)[15]	Worldwide	3b/4	208	RA	5mg/10mg	ADA, ETAN	4362	61	79
Kremer (2012) (NCT00413660)[16]	Worldwide	2b	24	RA	5mg/10mg	PBO	215	54	77
van der Heijde (2017) (NCT01786668)[17]	Worldwide	2	16	AxSpA	5mg/10mg	PBO	155	42	26
Fleischmann (2012) (NCT00550446)[18]	Worldwide	2b	24	RA	5mg/10mg	PBO, ADA	222	53	87
Nash (2021) (OPAL Balance LTE)[19]	Worldwide	LTE	192	PsA	5mg/10mg	PBO, ADA	897	49	54
Papp (2016) (OPT Pivotal LTE)[20]	Worldwide	LTE	143	PsO	5mg/10mg	PBO	2180	46	30
Sandborn (2022) (OCTAVE/RIVETING/LTE)[21]	Worldwide	LTE	406	UC	5mg/10mg	PBO	1355	41	NP
Cohen (2020) (ORAL Pooled P1-4/LTE)[22]	Worldwide	LTE	494	RA	5mg/10mg	PBO, ADA, MTX	8699	52	83
**Baricitinib**									
Dougados (2016) (RA-BUILD)[23]	Worldwide	3	24	RA	2mg/4mg	PBO	684	52	81
Genovese (2016) (RA-BEACON)[24]	Worldwide	3	24	RA	2mg/4mg	PBO	527	56	82
Keystone (2015) (NCT01185353)[25]	Worldwide	2b	24	RA	2mg/4mg	PBO	202	52	78
Taylor (2017) (RA-BEAM)[26]	Worldwide	3	52	RA	4mg	PBO, ADA	1305	54	77
Fleischmann (2017) (RA-BEGIN)[27]	Worldwide	3	52	RA	4mg	MTX	584	50	74
Li (2020) (RA-BALANCE)[28]	China, S.America	3	52	RA	4mg	PBO	290	49	90

Author (study year/name/reference)	Study location	Study phase	Study duration, weeks	Disease	Doses included in analyses, mg	Comparators	Number participants	Mean age, years	Female participants, %
Tanaka (2016) (NCT01469013)[29]	Japan	2b	16	RA	2mg/4mg	PBO	97	57	83
Simpson (2020) (BREEZE-AD1)[30]	Worldwide	3	16	AD	2mg/4mg	PBO	497	36	34
Simpson (2020) (BREEZE-AD2)[30]	Worldwide	3	16	AD	2mg/4mg	PBO	490	35	40
Bieber (2022) (BREEZE-AD4)[31]	Worldwide	3	52	AD	2mg/4mg	PBO	370	38	31
Simpson (2021) (BREEZE-AD5)[32]	N. America	3	16	AD	2mg	PBO	293	40	53
Reich (2020) (BREEZE-AD7)[33]	Worldwide	3	16	AD	2mg/4mg	PBO	328	34	34
Bieber (2020) (BREEZE AD Pooled/LTE)[34]	Worldwide	LTE	105	AD	2mg/4mg	PBO	3274	36	39
Taylor (2022) (RA-BEYOND Pooled/LTE)[35]	Worldwide	LTE	484	RA	2mg/4mg	PBO, ADA, MTX	5494	53	79
**Upadacitinib**									
van der Heijde (2019) (SELECT-AXIS 1)[36]	Worldwide	2/3	14	AxSpA	15mg	PBO	186	47	32
Fleischmann (2019) (SELECT-COMPARE)[37]	Worldwide	3	48	RA	15mg	PBO, ADA	1629	54	80
Smolen (2019) (SELECT-MONO)[38]	Worldwide	3	14	RA	15mg/30mg	MTX	648	54	80
van Vollenhoven (2019) (SELECT-EARLY)[39,40]	Worldwide	3	48	RA	15mg/30mg	MTX	945	53	76
Guttman-Yassky (2021) (MEASURE UP 1+2)[41,42]	Worldwide	3	52	AD	15mg/30mg	PBO	1889	34	44
McInnes (2021) (SELECT-PsA 1)[43-45]	Worldwide	3	56	PsA	15mg/30mg	PBO, ADA	2082	51	56
Mease (2021) (SELECT-PsA 2)[45-47]	Worldwide	3	56	PsA	15mg/30mg	PBO	810	53	53
Danese (2022) (U-ACHIEVE Maintenance)[48]	Worldwide	3	52	UC	15mg/30mg	PBO	451	41	39
Silverberg (2022) (AD Up)[49]	Worldwide	3	52	AD	15mg/30mg	PBO	1183	34	38
Burmester (2018) (SELECT-NEXT)[50]	Worldwide	3	12	RA	15mg/30mg	PBO	661	56	81
Genovese (2018) (SELECT-BEYOND)[51]	Worldwide	3	24	RA	15mg/30mg	PBO	498	57	84
Zeng (2021) (NCT02955212)[52]	China, S. Korea, Brazil	3	12	RA	15mg	PBO	338	52	80
Kameda (2020) (SELECT-SUNRISE)[53]	Japan	2b/3	12	RA	15mg/30mg	PBO	148	55	80
van der Heijde (2022) (SELECT-AXIS 2)[54]	Worldwide	3	14	AxSpA	15mg	PBO	420	42	27
Guttman-Yassky (2020) (NCT02925117)[55]	Worldwide	2b	16	AD	15mg/30mg	PBO	125	40	39
Katoh (2022) (RISING UP)[56]	Japan	3	24	AD	15mg/30mg	PBO	320	35	25
Cohen (2020) (SELECT Pooled Phase 3/LTE)[57]	Worldwide	3/LTE	130	RA	15mg/30mg	PBO, ADA, MTX	5985	55	80
van der Heijde (2022) (SELECT-AXIS 1 LTE)[58]	Worldwide	LTE	104	AxSpA	15mg	PBO	271	47	32
Zeng (2021) (NCT02955212 LTE)[59]	China, S. Korea, Brazil	LTE	64	RA	15mg	PBO	491	52	80
D'Haens (2021) (CELEST LTE)[60]	Worldwide	LTE	130	Crohn's	15mg/30mg	PBO	144	41	55
Kameda (2021) (SELECT-SUNRISE LTE)[61]	Japan	LTE	84	RA	15mg/30mg	PBO	236	55	80
Katoh (2022) (RISING UP LTE)[62]	Japan	LTE	112	AD	15mg/30mg	PBO	359	35	25
**Filgotinib**									

Author (study year/name/reference)	Study location	Study phase	Study duration, weeks	Disease	Doses included in analyses, mg	Comparators	Number participants	Mean age, years	Female participants, %
Genovese (2019) (FINCH 2)[63]	Worldwide	3	24	RA	100mg/200mg	PBO	449	56	80
Gilead (2022) (DIVERGENCE 2)[64]	N. America, Europe	2	24	Crohn's	100mg/200mg	PBO	57	40	45
Westhovens (2017) (DARWIN 1)[65]	Worldwide	2b	24	RA	100mg/200mg	PBO	408	54	81
Kavanaugh (2017) (DARWIN 2)[66]	Worldwide	2b	24	RA	100mg/200mg	PBO	226	53	81
Feagan (2021) (SELECTION)[67]	Worldwide	2b/3	58	UC	100mg/200mg	PBO	1348	43	43
Westhovens (2021) (FINCH 3)[68]	Worldwide	3	52	RA	100mg/200mg	MTX	1249	53	80
Combe (2021) (FINCH 1)[69]	Worldwide	3	52	RA	100mg/200mg	PBO, ADA	1759	53	82
Mease (2018) (EQUATOR)[70]	Europe	2	20	PsA	200mg	PBO	131	49	55
van der Heijde (2018) (TORTUGA)[71]	Europe	2	16	AxSpA	200mg	PBO	116	41	22
Mease (2020) (EQUATOR 2 LTE)[72]	Europe	LTE	100	PsA	200mg	PBO	188	NP	NP
Winthrop (2022) (Pooled FINCH/DARWIN + LTE)[73]	Worldwide	2/3/LTE	354	RA	100mg/200mg	PBO	5215	53	80
Schreiber (2022) (SELECTION LTE)[74]	Worldwide	LTE	82	UC	100mg/200mg	PBO	1532	NP	NP
**Peficitinib**									
Takeuchi (2019) (RAJ 4)[75]	Japan	3	52	RA	100mg/150mg	PBO	519	57	70
Tanaka (2019) (RAJ 3)[76]	Japan	3	52	RA	100mg/150mg	PBO, ETAN	597	55	75
Takeuchi (2016) (RAJ 1)[77]	Japan	2b	16	RA	100mg/150mg	PBO	169	52	82
Sands (2018) (NCT01959282)[78]	Worldwide	2	36	UC	150mg	PBO	131	33	50
Kivitz (2017) (NCT01554696)[79]	N./C. America, Europe	2b	16	RA	100mg/150mg	PBO	234	54	82
Genovese (2017) (NCT01565655)[80]	N./C. America, Europe	2b	16	RA	100mg/150mg	PBO	173	55	83
Takeuchi (2021) (RAJ 2 LTE)[81]	Japan	LTE	369	RA	100mg/150mg	PBO, ETAN	1334	56	73

For study location, worldwide refers to a study that was performed in 3 or more continents. Mean age and the proportion of female participants are shown for pooled JAK inhibitor groups of each study, where available. Study duration refers to the maximum duration of follow-up included in meta-analyses. Number of participants refers to the total participant numbers included in these analyses from all relevant study arms; for LTE studies not reporting comparator data, then comparator data from the original RCTs was incorporated. LTE: long-term extension study; RA: rheumatoid arthritis; PsA: psoriatic arthritis; PsO: psoriasis; AxSpA: axial spondyloarthritis; UC: ulcerative colitis; AD: atopic dermatitis; PBO: placebo; ADA: adalimumab; ETAN: etanercept; MTX: methotrexate; NP: not provided.

Supplementary Table S2. Risk of bias assessment for included RCTs.

Author (study year and name)	Random sequence generation	Allocation concealment	Blinding patients and personnel	Blinding outcome assessment	Incomplete outcome data	Selective reporting
**Tofacitinib**						
Kremer (2013) (ORAL Sync)						
Gladman (2017) (OPAL Beyond)						
Mease (2017) (OPAL Broaden)						
Sandborn (2017) (OCTAVE Sustain)						
Deodhar (2021) (NCT03502616)						
Fleischmann (2012) (ORAL Solo)						
van Vollenhoven (2012) (ORAL Standard)						
Lee (2014) (ORAL Start)						
Fleischmann (2017) (ORAL Strategy)						
Papp (2015) (OPT Pivotal 1+2)						
Panés (2017) (NCT01393626)						
Burmester (2013) (ORAL Step)						
Zhang (2017) (NCT01815424)						
van der Heijde (2019) (ORAL Scan)						
Ytterberg (2022) (ORAL Surveillance)						
Kremer (2012) (NCT00413660)						
van der Heijde (2017) (NCT01786668)						
Fleischmann (2012) (NCT00550446)						
**Baricitinib**						
Dougados (2016) (RA-BUILD)						
Genovese (2016) (RA-BEACON)						
Keystone (2015) (NCT01185353)						
Taylor (2017) (RA-BEAM)						
Fleischmann (2017) (RA-BEGIN)						
Li (2020) (RA-BALANCE)						
Tanaka (2016) (NCT01469013)						

Author (study year and name)	Random sequence generation	Allocation concealment	Blinding patients and personnel	Blinding outcome assessment	Incomplete outcome data	Selective reporting
Simpson (2020) (BREEZE-AD1/AD2)						
Bieber (2022) (BREEZE-AD4)						
Simpson (2021) (BREEZE-AD5)						
Reich (2020) (BREEZE-AD7)						
**Upadacitinib**						
van der Heijde (2019) (SELECT-AXIS 1)						
Fleischmann (2019) (SELECT-COMPARE)						
Smolen (2019) (SELECT-MONO)						
van Vollenhoven (2019) (SELECT-EARLY)						
Guttman-Yassky (2021) (MEASURE UP 1+2)						
McInnes (2021) (SELECT-PsA 1)						
Mease (2021) (SELECT-PsA 2)						
Danese (2022) (U-ACHIEVE)						
Silverberg (2022) (AD Up)						
Burmester (2018) (SELECT-NEXT)						
Genovese (2018) (SELECT-BEYOND)						
Zeng (2021) (NCT02955212)						
Kameda (2020) (SELECT-SUNRISE)						
van der Heijde (2022) (SELECT-AXIS 2)						
Guttman-Yassky (2020) (NCT02925117)						
Katoh (2022) (RISING UP)						
**Filgotinib**						
Genovese (2019) (FINCH 2)						
Gilead (2022) (DIVERGENCE 2)						
Westhovens (2017) (DARWIN 1)						
Kavanaugh (2017) (DARWIN 2)						
Feagan (2021) (SELECTION)						
Westhovens (2021) (FINCH 3)						

Author (study year and name)	Random sequence generation	Allocation concealment	Blinding patients and personnel	Blinding outcome assessment	Incomplete outcome data	Selective reporting
Combe (2021) (FINCH 1)						
Mease (2018) (EQUATOR)						
Van der Heijde (2018) (TORTUGA)						
**Peficitinib**						
Takeuchi (2019) (RAJ 4)						
Tanaka (2019) (RAJ 3)						
Takeuchi (2016) (RAJ 1)						
Sands (2018) (NCT01959282)						
Kivitz (2017) (NCT01554696)						
Genovese (2017) (NCT01565655)						

Risk of bias was assessed for each domain using the Cochrane Risk of Bias-2 tool. Green = low risk of bias; yellow = some concerns for bias; red = high risk of bias.

Supplementary Table S3. Surface under the cumulative ranking curve (SUCRA) method to rank malignancy risk between treatments from network meta-analyses.

For RCT data

	Treatment			
**Rank**	**JAKi**	**TNFi**	**Methotrexate**	**Placebo**
Best	0.2	92.7	6.9	0.2
Second	68.6	7.2	16.8	7.4
Third	29.0	0.1	32.4	38.5
Worst	2.2	0.0	43.9	53.9
Mean Rank	2.3	1.1	3.1	3.5
SUCRA	0.6	1.0	0.3	0.2

For combined RCT and LTE data

	Treatment			
**Rank**	**JAKi**	**TNFi**	**Methotrexate**	**Placebo**
Best	0.0	84.2	7.6	8.2
Second	13.4	13.8	29.1	43.7
Third	46.3	2.0	22.5	29.2
Worst	40.3	0.0	40.8	18.9
Mean Rank	3.3	1.2	3.0	2.6
SUCRA	0.2	0.9	0.3	0.5

Each treatment was ranked based upon the estimated probability (%) of it causing the least number of malignancies under the cumulative ranking curve (SUCRAs). This was derived from network meta-analyses of RCT data (top) and RCT/LTE data (bottom). Higher SUCRA values indicate a greater likelihood of a given treatment causing the least number of malignancies, such that when the SUCRA value is 1, the treatment is certain to be the best, and when it is 0, it is certain to be the worst.

Supplementary Table S4. Meta-regression summary table of beta-coefficients for age and sex on incidence rate ratios for all malignancies between JAKi and comparators.

Variable	Comparison	Beta coefficient (95% CI)	p-value
Mean age, years	JAKi vs. placebo	-0.008 (-0.085 to 0.069)	0.84
	JAKi vs. TNFi	0.036 (-0.043 to 0.115)	0.32
	JAKi vs. methotrexate	-0.23 (-1.06 to 0.59)	0.44
Female sex	JAKi vs. placebo	-0.75 (-3.33 to 1.83)	0.56
	JAKi vs. TNFi	-0.57 (-5.24 to 4.11)	0.79
	JAKi vs. methotrexate	-10.3 (-69.8 to 49.3)	0.62

Outputs are shown from random effects meta-regression, exploring associations between age and sex differences of JAKi and comparator arms of RCTs and incidence rate ratios for all malignancies including non-melanomatous skin cancers. JAKi: Janus kinase inhibitor; TNFi: Tumour Necrosis Factor-alpha inhibitor.

Supplementary Data S1: Literature search strategy

Databases searched:

Embase: Database inception to 9 December 2022; Ovid MEDLINE: Database inception to 9 December 2022; Cochrane library: Database inception to 9 December 2022.

Search terms:

tofacitinib OR baricitinib OR upadacitinib OR filgotinib OR peficitinib OR xeljanz OR jakvinus OR tasocitinib OR olumiant OR smyraf OR rinvoq OR jyseleca OR CP-690 OR CP-690550 OR ABT-494 OR ABT494 OR G-146034 OR G146034 OR GLPG-0634 OR GLPG0634 OR ASP015K OR JNJ-54781532

AND

rheumatoid OR psoriatic arthritis OR psoriasis OR ankylosing spondylitis OR axial spondyloarthritis OR ulcerative colitis OR crohn OR crohn's OR crohns OR atopic dermatitis OR eczema

Search limitations:

English language studies; clinical trials performed in humans

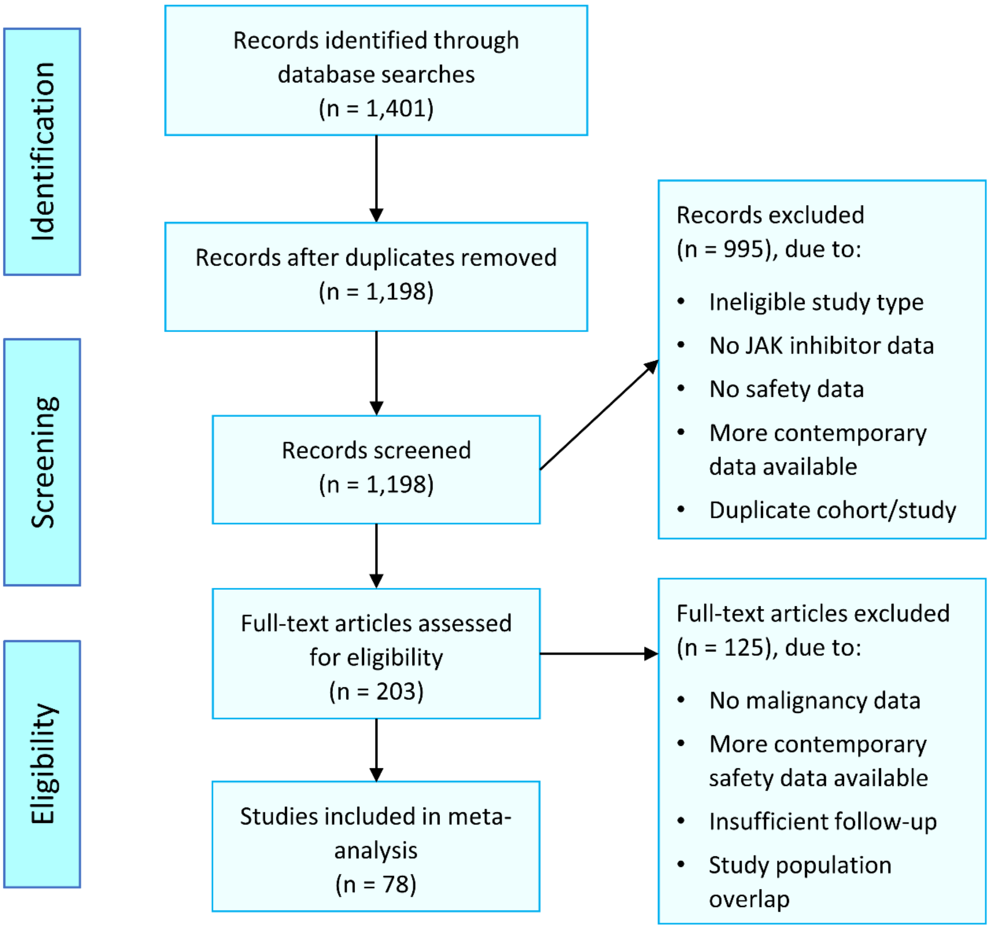

Supplementary Figure S1: Flowchart of studies identified in the systematic literature search

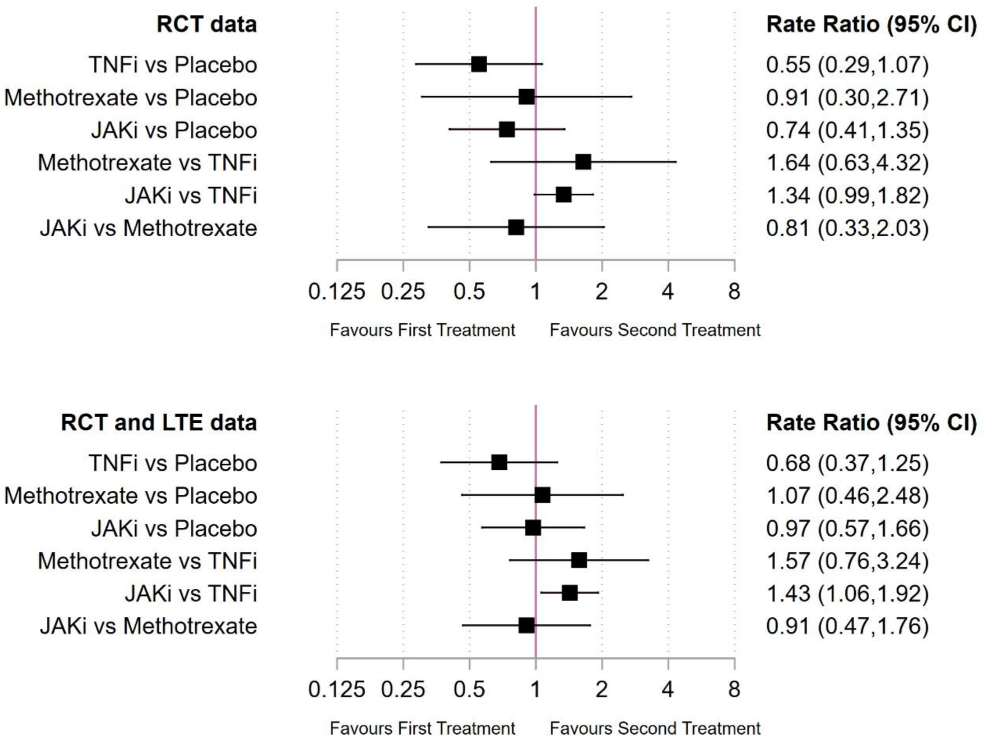

Supplementary Figure S2. Network meta-analysis of the risk of all malignancies excluding non-melanomatous skin cancers, comparing study groups in eligible RCTs (top panel) and combined RCT and LTE studies (bottom panel); expressed as incidence rate ratios with 95% CIs and depicted graphically as a forest plot. JAKi: Janus kinase inhibitor; TNFi: Tumour Necrosis Factor-alpha inhibitor**;** RCT: randomised clinical trial; LTE: long-term extension.

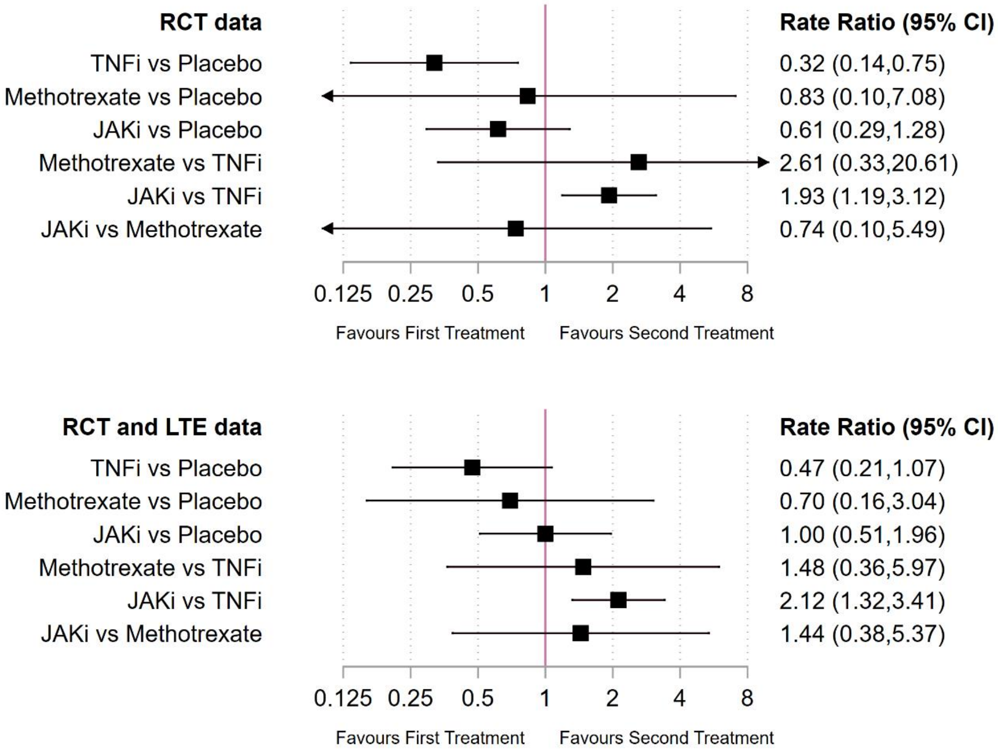

Supplementary Figure S3. Network meta-analysis of the risk of non-melanomatous skin cancers between study groups in eligible RCTs (top panel) and combined RCT and LTE studies (bottom panel); expressed as incidence rate ratios with 95% CIs and depicted graphically as a forest plot. JAKi: Janus kinase inhibitor; TNFi: Tumour Necrosis Factor-alpha inhibitor**;** RCT: randomised clinical trial; LTE: long-term extension.

Study	Treatment Events	Treatment Exposure	Control Events	Control Exposure	Incidence Rate Ratio with 95% CI	Weight (%)
BREEZE AD Pooled/LTE (Baricitinib 2mg)	11	2,247	3	212	0.34 [0.10, 1.19]	11.56
RA-BEYOND Pooled/LTE (Baricitinib 4mg/2mg)	189	14,744	3	450	1.85 [0.61, 5.67]	14.47
DIVERGENCE 2 (Filgotinib 200mg/100mg)	0	12	0	3	0.24 [0.00, 1422]	0.24
EQUATOR 2 LTE (Filgotinib 200mg)	1	262	0	20	0.83 [0.00, 530]	0.43
FINCH/DARWIN Pooled/LTE (Filgotinib 200mg/100mg)	69	8,085	3	268	0.74 [0.24, 2.3]	14.14
SELECTION LTE (Filgotinib 200mg/100mg)	24	1,605	1	102	1.38 [0.21, 9.25]	5.01
TORTUGA (Filgotinib 200mg)	0	13	0	12	0.95 [0.00, 5851]	0.24
NCT01554696 (Peficitinib 150mg/100mg)	0	34	0	16	0.45 [0.00, 2849]	0.24
NCT01565655 (Peficitinib 150mg/100mg)	0	26	0	11	0.43 [0.00, 2706]	0.24
NCT01959282 (Peficitinib 150mg)	2	49	1	15	0.59 [0.06, 5.52]	3.62
RAJ 2 LTE (Peficitinib 150mg/100mg)	27	2,170	1	95	1.08 [0.16, 7.18]	5.04
NCT01393626 (Tofacitinib 10mg/5mg)	1	72	0	33	5.04 [0.01, 3223]	0.43
NCT01786668 (Tofacitinib 10mg/5mg)	0	30	0	14	0.48 [0.00, 3027]	0.24
NCT01815424 (Tofacitinib 10mg/5mg)	2	211	0	24	2.35 [0.00, 1319]	0.45
NCT03502616 (Tofacitinib 5mg)	0	187	0	40	0.22 [0.00, 1374]	0.24
OCTAVE/RIVETING/LTE (Tofacitinib 10mg/5mg)	48	3,000	2	100	0.77 [0.20, 3.03]	9.68
OPAL Balance LTE (Tofacitinib 10mg/5mg)	27	1,702	0	53	8.34 [0.02, 4121]	0.47
OPT Pivotal LTE (Tofacitinib 10mg/5mg)	50	2,705	0	87	15.93 [0.03, 7854]	0.47
ORAL Pooled P1-4/LTE (Tofacitinib 10mg/5mg)	306	22,875	2	218	1.38 [0.36, 5.34]	9.92
AD Up (Upadacitinib 30mg/15mg)	4	1,045	0	87	3.39 [0.01, 1789]	0.46
CELEST LTE (Upadacitinib 30mg/15mg)	2	197	0	8	0.90 [0.00, 493]	0.46
MEASURE UP 1+2 (Upadacitinib 30mg/15mg)	16	1,932	0	149	12.32 [0.02, 6164]	0.47
NCT02925117 (Upadacitinib 30mg/15mg)	0	23	0	6	0.27 [0.00, 1671]	0.24
NCT02955212 LTE (Upadacitinib 15mg)	3	335	0	35	3.26 [0.01, 1753]	0.46
RISING UP LTE (Upadacitinib 30mg/15mg)	1	577	0	27	0.51 [0.00, 329]	0.43
SELECT Pooled (Upadacitinib 30mg/15mg)	66	4,020	2	257	2.00 [0.51, 7.84]	9.67
SELECT-AXIS 1 LTE (Upadacitinib 15mg)	1	309	0	24	0.86 [0.00, 549]	0.43
SELECT-AXIS 2 (Upadacitinib 15mg)	0	55	1	55	0.09 [0.00, 58.6]	0.43
SELECT-PSA 1/2 (Upadacitinib 30mg/15mg)	39	2,505	1	269	3.77 [0.57, 25]	5.06
SELECT-SUNRISE LTE (Upadacitinib 30mg/15mg)	2	156	0	11	1.46 [0.00, 808]	0.45
U-ACHIEVE (Upadacitinib 30mg/15mg)	5	381	1	128	1.55 [0.20, 12.1]	4.30
**Overall**					1.12 [0.73, 1.71]	

Heterogeneity: $\tau^2 = 0.00$, $I^2 = 0.00\%$, $H^2 = 1.00$

Test of $\theta = 0$: $z = 0.51$, $p = 0.61$

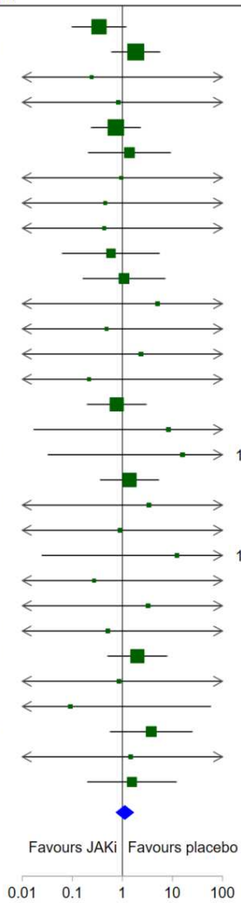

Fixed continuity correction of 0.1 applied to studies with zero events

Supplementary Figure S4. Pairwise meta-analysis of the risk of all malignancies including non-melanomatous skin cancers between JAKi and placebo groups in combined RCT and LTE data; expressed as incidence rate ratios with 95% CIs and depicted graphically as a forest plot. The relative weighting of each study from a random-effects model is shown. A fixed continuity correction of 0.1 was used for studies with zero events. Heterogeneity between studies was assessed using I² statistics. Further details and references for included studies are provided within Supplementary Table S1. JAKi: Janus kinase inhibitor; RCT: randomised clinical trial; LTE: long-term extension.

Study	Treatment Events	Treatment Exposure	Control Events	Control Exposure	Incidence Rate Ratio with 95% CI	Weight (%)
**Tofacitinib**						
NCT01393626 (Tofacitinib 10mg/5mg)	1	72	0	33	5.04 [0.01, 3223]	0.43
NCT01786668 (Tofacitinib 10mg/5mg)	0	30	0	14	0.48 [0.00, 3027]	0.24
NCT01815424 (Tofacitinib 10mg/5mg)	2	211	0	24	2.35 [0.00, 1319]	0.45
NCT03502616 (Tofacitinib 5mg)	0	187	0	40	0.22 [0.00, 1374]	0.24
OCTAVE/RIVETING/LTE (Tofacitinib 10mg/5mg)	48	3,000	2	100	0.77 [0.20, 3.03]	9.68
OPAL Balance LTE (Tofacitinib 10mg/5mg)	27	1,702	0	53	8.34 [0.02, 4121]	0.47
OPT Pivotal LTE (Tofacitinib 10mg/5mg)	50	2,705	0	87	15.93 [0.03, 7854]	0.47
ORAL Pooled P1-4/LTE (Tofacitinib 10mg/5mg)	306	22,875	2	218	1.38 [0.36, 5.34]	9.92
Heterogeneity: $\tau^2 = 0.00$, $I^2 = 0.00\%$, $H^2 = 1.00$					1.18 [0.47, 2.92]	
Test of $\theta_i = \theta_j$: Q(7) = 1.91, p = 0.96						
Test of $\theta = 0$: z = 0.35, p = 0.73						
**Baricitinib**						
BREEZE AD Pooled/LTE (Baricitinib 2mg)	11	2,247	3	212	0.34 [0.10, 1.19]	11.56
RA-BEYOND Pooled/LTE (Baricitinib 4mg/2mg)	189	14,744	3	450	1.85 [0.61, 5.67]	14.47
Heterogeneity: $\tau^2 = 1.07$, $I^2 = 74.40\%$, $H^2 = 3.91$					0.81 [0.16, 4.27]	
Test of $\theta_i = \theta_j$: Q(1) = 3.91, p = 0.05						
Test of $\theta = 0$: z = -0.24, p = 0.81						
**Upadacitinib**						
AD Up (Upadacitinib 30mg/15mg)	4	1,045	0	87	3.39 [0.01, 1789]	0.46
CELEST LTE (Upadacitinib 30mg/15mg)	2	197	0	8	0.90 [0.00, 493]	0.46
MEASURE UP 1+2 (Upadacitinib 30mg/15mg)	16	1,932	0	149	12.32 [0.02, 6164]	0.47
NCT02925117 (Upadacitinib 30mg/15mg)	0	23	0	6	0.27 [0.00, 1671]	0.24
NCT02955212 LTE (Upadacitinib 15mg)	3	335	0	35	3.26 [0.01, 1753]	0.46
RISING UP LTE (Upadacitinib 30mg/15mg)	1	577	0	27	0.51 [0.00, 329]	0.43
SELECT Pooled (Upadacitinib 30mg/15mg)	66	4,020	2	257	2.00 [0.51, 7.84]	9.67
SELECT-AXIS 1 LTE (Upadacitinib 15mg)	1	309	0	24	0.86 [0.00, 549]	0.43
SELECT-AXIS 2 (Upadacitinib 15mg)	0	55	1	55	0.09 [0.00, 58.6]	0.43
SELECT-PSA 1/2 (Upadacitinib 30mg/15mg)	39	2,505	1	269	3.77 [0.57, 25]	5.06
SELECT-SUNRISE LTE (Upadacitinib 30mg/15mg)	2	156	0	11	1.46 [0.00, 808]	0.45
U-ACHIEVE (Upadacitinib 30mg/15mg)	5	381	1	128	1.55 [0.20, 12.1]	4.30
Heterogeneity: $\tau^2 = 0.00$, $I^2 = 0.00\%$, $H^2 = 1.00$					2.01 [0.83, 4.9]	
Test of $\theta_i = \theta_j$: Q(11) = 2.25, p = 1.00						
Test of $\theta = 0$: z = 1.54, p = 0.12						
**Filgotinib**						
DIVERGENCE 2 (Filgotinib 200mg/100mg)	0	12	0	3	0.24 [0.00, 1422]	0.24
EQUATOR 2 LTE (Filgotinib 200mg)	1	262	0	20	0.83 [0.00, 530]	0.43
FINCH/DARWIN Pooled/LTE (Filgotinib 200mg/100mg)	69	8,085	3	268	0.74 [0.24, 2.3]	14.14
SELECTION LTE (Filgotinib 200mg/100mg)	24	1,605	1	102	1.38 [0.21, 9.25]	5.01
TORTUGA (Filgotinib 200mg)	0	13	0	12	0.95 [0.00, 5851]	0.24
Heterogeneity: $\tau^2 = 0.00$, $I^2 = 0.00\%$, $H^2 = 1.00$					0.86 [0.33, 2.22]	
Test of $\theta_i = \theta_j$: Q(4) = 0.39, p = 0.98						
Test of $\theta = 0$: z = -0.31, p = 0.75						
**Peficitinib**						
NCT01554696 (Peficitinib 150mg/100mg)	0	34	0	16	0.45 [0.00, 2849]	0.24
NCT01565655 (Peficitinib 150mg/100mg)	0	26	0	11	0.43 [0.00, 2706]	0.24
NCT01959282 (Peficitinib 150mg)	2	49	1	15	0.59 [0.06, 5.52]	3.62
RAJ 2 LTE (Peficitinib 150mg/100mg)	27	2,170	1	95	1.08 [0.16, 7.18]	5.04
Heterogeneity: $\tau^2 = 0.00$, $I^2 = 0.00\%$, $H^2 = 1.00$					0.81 [0.20, 3.31]	
Test of $\theta_i = \theta_j$: Q(3) = 0.20, p = 0.98						
Test of $\theta = 0$: z = -0.29, p = 0.77						
**Overall**					1.12 [0.73, 1.71]	
Heterogeneity: $\tau^2 = 0.00$, $I^2 = 0.00\%$, $H^2 = 1.00$						
Test of $\theta = 0$: z = 0.51, p = 0.61						
Test of group differences: $Q_b(4) = 2.28$, p = 0.68						

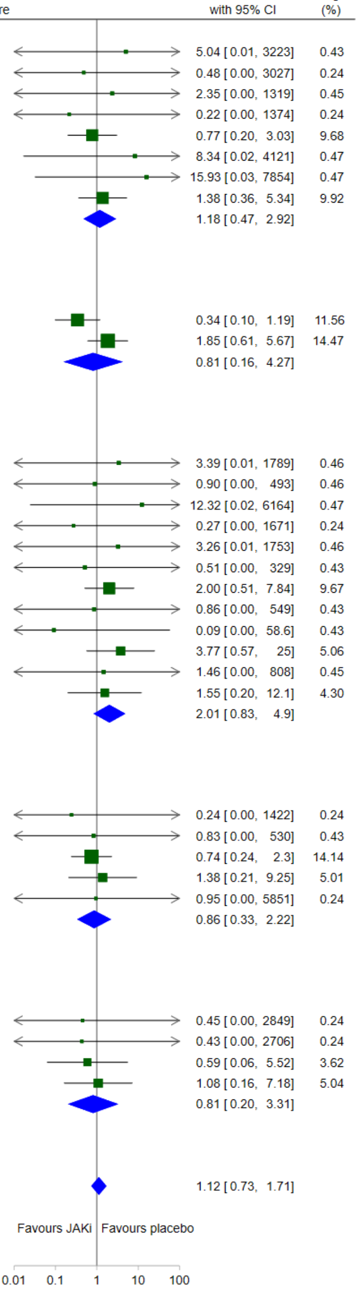

Supplementary Figure S5. Pairwise meta-analysis of the risk of all malignancies including non-melanomatous skin cancers between individual JAKi medications and placebo groups of combined RCT and LTE data; expressed as incidence rate ratios with 95% CIs and depicted graphically as a forest plot. Exposure is reported in person-years. The relative weighting of each study from a random-effects model is shown. A fixed continuity correction of 0.1 was used for studies with zero events. Heterogeneity between studies was assessed using I² statistics. Further details and references for included studies are provided within Supplementary Table S1. JAKi: Janus kinase inhibitor.

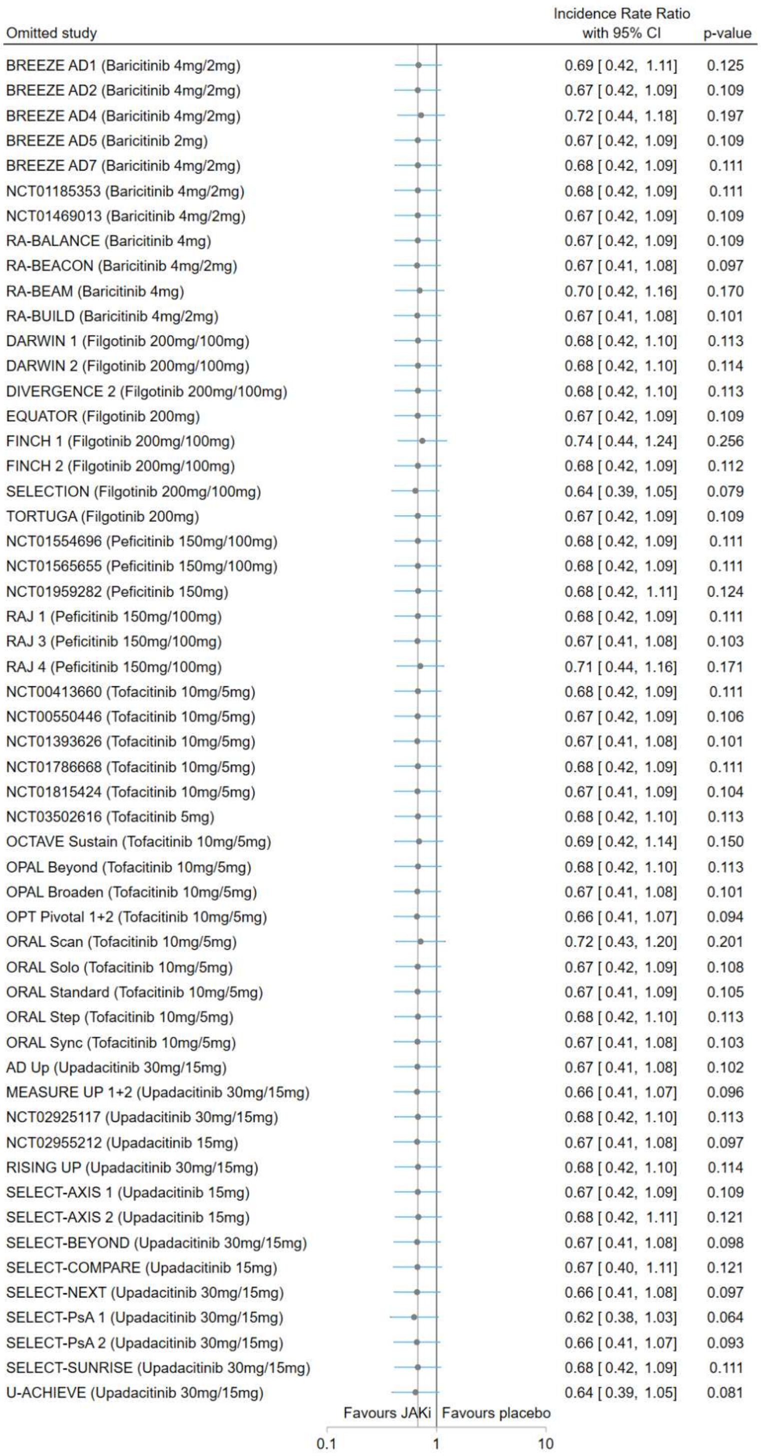

Supplementary Figure S6. Sensitivity analysis demonstrating the influence of individual studies on the meta-analysis results for all malignancy events, comparing JAKi and placebo groups of eligible RCTs. The effect estimates (incidence rate ratio, 95% CIs, p-values) provided on each row correspond to the pairwise meta-analysis results when excluding that study. A fixed continuity correction of 0.1 was used for studies with zero events. JAKi: Janus kinase inhibitor.

Omitted study	Incidence Rate Ratio with 95% CI	p-value
BREEZE AD Pooled/LTE (Baricitinib 2mg)	1.30 [0.83, 2.05]	0.249
RA-BEYOND Pooled/LTE (Baricitinib 4mg/2mg)	1.03 [0.65, 1.62]	0.914
DIVERGENCE 2 (Filgotinib 200mg/100mg)	1.12 [0.73, 1.72]	0.599
EQUATOR 2 LTE (Filgotinib 200mg)	1.12 [0.73, 1.71]	0.607
FINCH/DARWIN Pooled/LTE (Filgotinib 200mg/100mg)	1.20 [0.76, 1.89]	0.447
SELECTION LTE (Filgotinib 200mg/100mg)	1.10 [0.71, 1.71]	0.655
TORTUGA (Filgotinib 200mg)	1.12 [0.73, 1.71]	0.609
NCT01554696 (Peficitinib 150mg/100mg)	1.12 [0.73, 1.71]	0.603
NCT01565655 (Peficitinib 150mg/100mg)	1.12 [0.73, 1.71]	0.603
NCT01959282 (Peficitinib 150mg)	1.14 [0.74, 1.76]	0.542
RAJ 2 LTE (Peficitinib 150mg/100mg)	1.12 [0.72, 1.73]	0.613
NCT01393626 (Tofacitinib 10mg/5mg)	1.11 [0.72, 1.70]	0.632
NCT01786668 (Tofacitinib 10mg/5mg)	1.12 [0.73, 1.71]	0.604
NCT01815424 (Tofacitinib 10mg/5mg)	1.11 [0.73, 1.71]	0.622
NCT03502616 (Tofacitinib 5mg)	1.12 [0.73, 1.72]	0.598
OCTAVE/RIVETING/LTE (Tofacitinib 10mg/5mg)	1.16 [0.74, 1.82]	0.510
OPAL Balance LTE (Tofacitinib 10mg/5mg)	1.11 [0.72, 1.70]	0.642
OPT Pivotal LTE (Tofacitinib 10mg/5mg)	1.10 [0.72, 1.69]	0.652
ORAL Pooled P1-4/LTE (Tofacitinib 10mg/5mg)	1.09 [0.70, 1.71]	0.703
AD Up (Upadacitinib 30mg/15mg)	1.11 [0.73, 1.70]	0.627
CELEST LTE (Upadacitinib 30mg/15mg)	1.12 [0.73, 1.71]	0.608
MEASURE UP 1+2 (Upadacitinib 30mg/15mg)	1.10 [0.72, 1.69]	0.648
NCT02925117 (Upadacitinib 30mg/15mg)	1.12 [0.73, 1.72]	0.600
NCT02955212 LTE (Upadacitinib 15mg)	1.11 [0.73, 1.70]	0.627
RISING UP LTE (Upadacitinib 30mg/15mg)	1.12 [0.73, 1.72]	0.600
SELECT Pooled (Upadacitinib 30mg/15mg)	1.05 [0.67, 1.64]	0.831
SELECT-AXIS 1 LTE (Upadacitinib 15mg)	1.12 [0.73, 1.71]	0.607
SELECT-AXIS 2 (Upadacitinib 15mg)	1.13 [0.74, 1.73]	0.576
SELECT-PSA 1/2 (Upadacitinib 30mg/15mg)	1.05 [0.68, 1.62]	0.837
SELECT-SUNRISE LTE (Upadacitinib 30mg/15mg)	1.12 [0.73, 1.71]	0.615
U-ACHIEVE (Upadacitinib 30mg/15mg)	1.10 [0.71, 1.70]	0.665

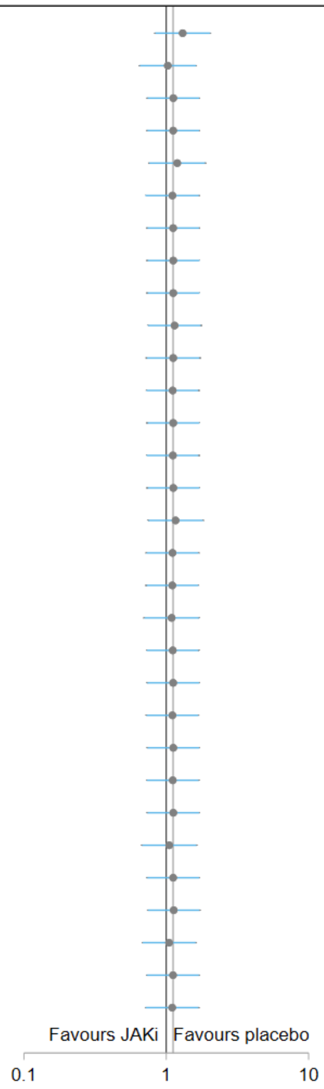

Supplementary Figure S7. Sensitivity analysis demonstrating the influence of individual studies on pairwise meta-analysis results for all malignancy events, comparing JAKi with placebo groups of eligible RCT and LTE studies. The effect estimates (incidence rate ratio, 95% CIs, p-values) provided on each row correspond to the pairwise meta-analysis results when excluding that study. A fixed continuity correction of 0.1 was used for studies with zero events. JAKi: Janus kinase inhibitor.

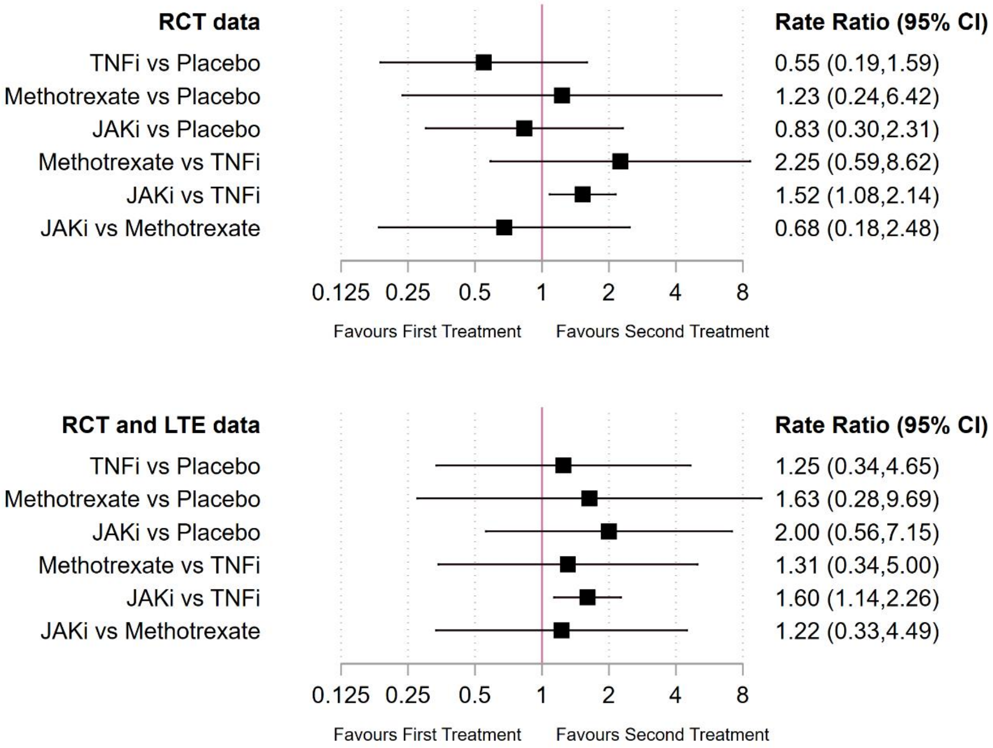

Supplementary Figure S8. Sensitivity network meta-analysis showing the risk of all malignancies, excluding those that occurred within the first 6 months of treatment, in RCTs (top panel) and combined RCT and LTE studies (bottom panel) that reported these data; expressed as incidence rate ratios with 95% CIs and depicted graphically as a forest plot. Data on timing of malignancies were available for 51/62 RCT studies and 6/16 LTE studies. JAKi: Janus kinase inhibitor; TNFi: Tumour Necrosis Factor-alpha inhibitor**;** RCT: randomised clinical trial; LTE: long-term extension.

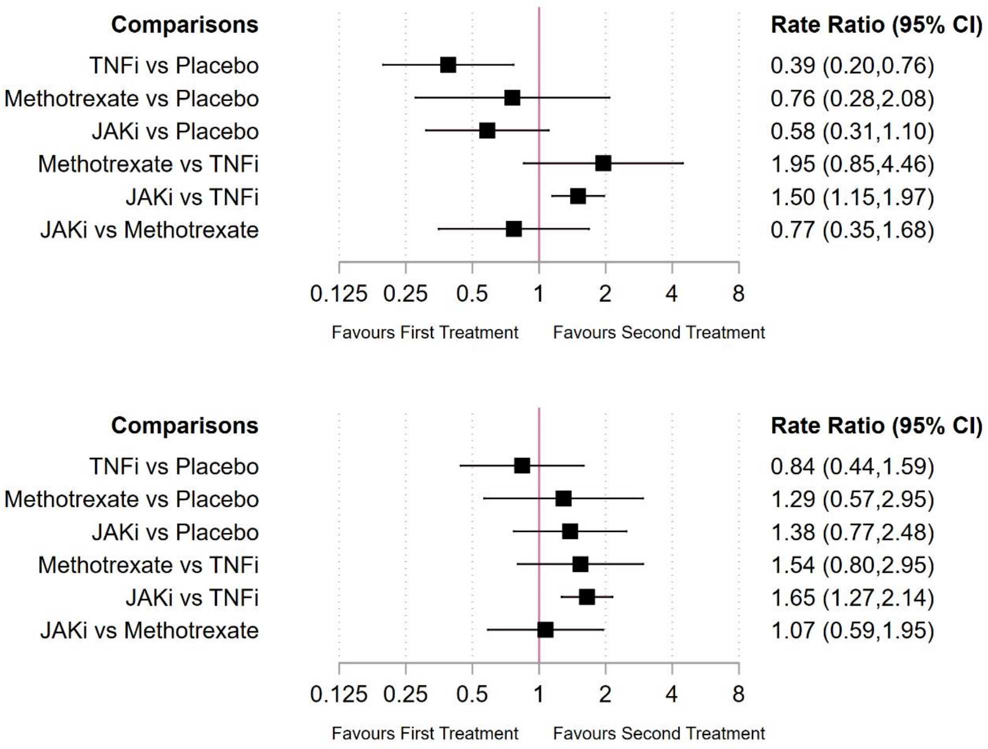

Supplementary Figure S9. Sensitivity network meta-analysis showing the risk of all malignancies including non-melanomatous skin cancers for participants with rheumatoid arthritis in RCTs (top panel) and combined RCT and LTE studies (bottom panel); expressed as incidence rate ratios with 95% CIs and depicted graphically as a forest plot. JAKi: Janus kinase inhibitor; TNFi: Tumour Necrosis Factor-alpha inhibitor**;** RCT: randomised clinical trial; LTE: long-term extension.

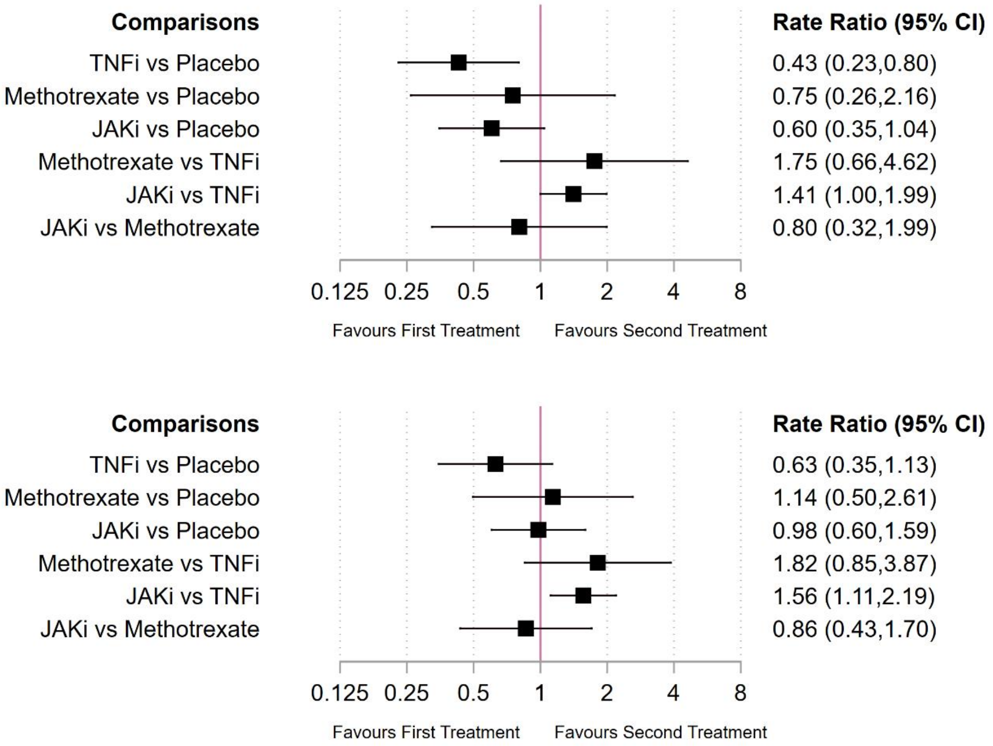

Supplementary Figure S10. Sensitivity network meta-analysis showing the risk of all malignancies including non-melanomatous skin cancers in RCTs (top panel) and combined RCT and LTE studies (bottom panel), excluding tofacitinib 10mg twice daily and upadacitinib 30mg once daily doses; expressed as incidence rate ratios with 95% CIs and depicted graphically as a forest plot. JAKi: Janus kinase inhibitor; TNFi: Tumour Necrosis Factor-alpha inhibitor**;** RCT: randomised clinical trial; LTE: long-term extension.

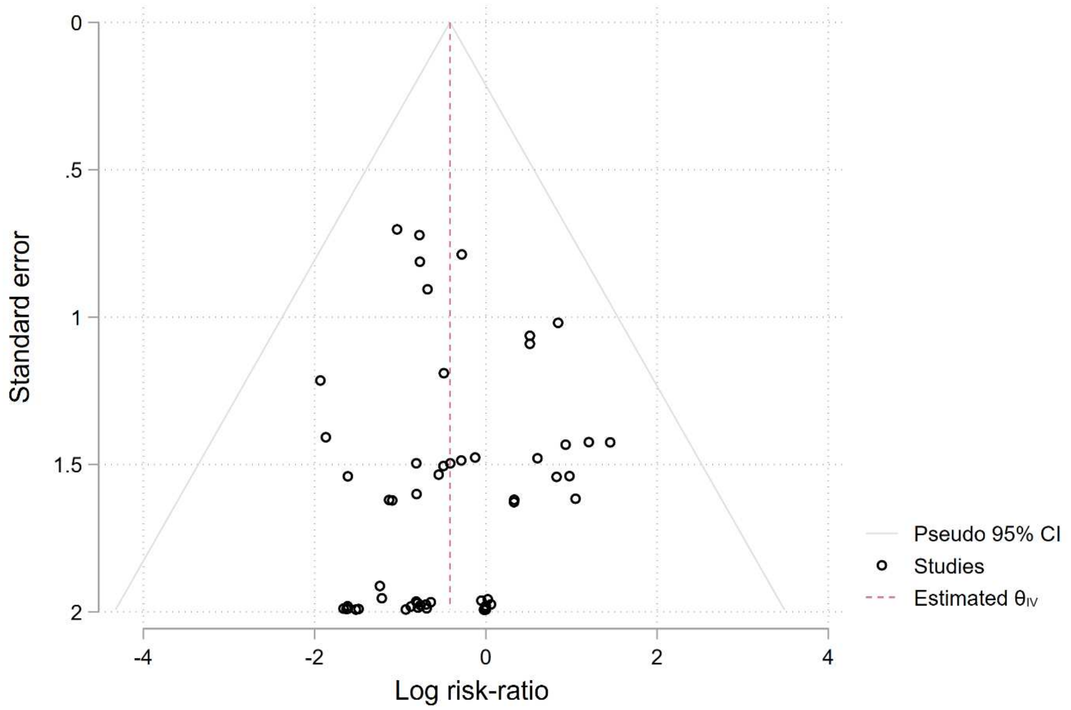

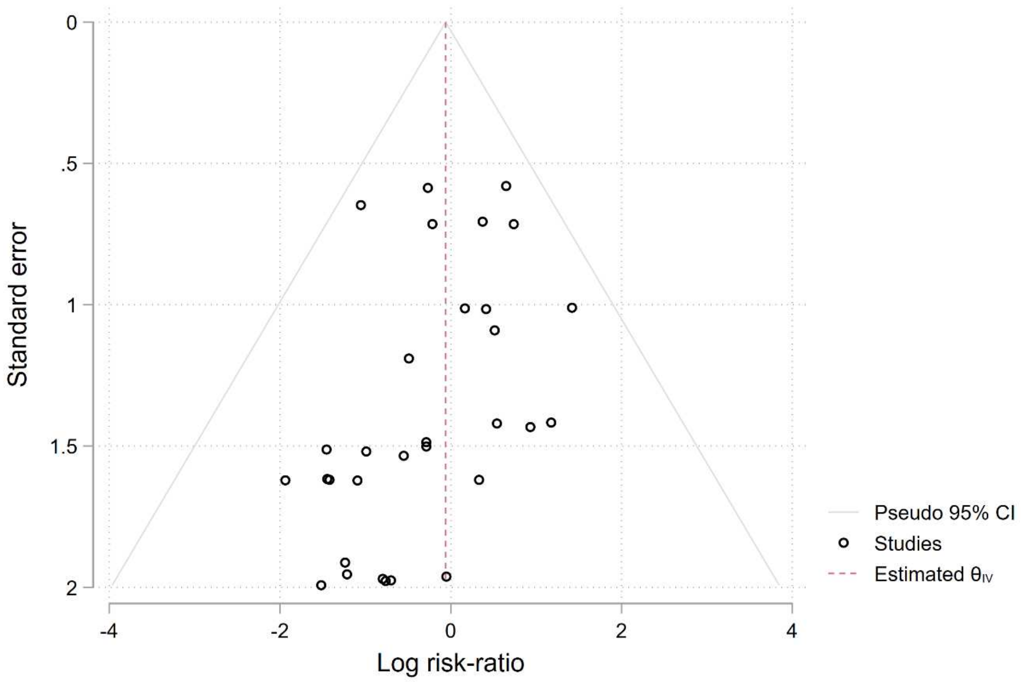

Supplementary Figure S11. Funnel plots, assessing potential publication bias in pairwise meta-analysis, comparing JAKi and placebo groups of eligible RCTs (top panel) and combined RCT and LTE data (bottom panel.

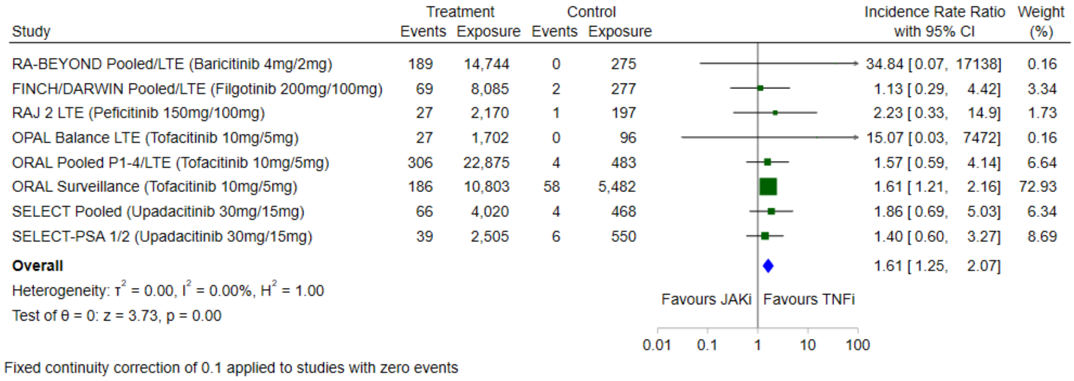

Supplementary Figure S12. Pairwise meta-analysis of the risk of all malignancies including non-melanomatous skin cancers between JAKi and TNFi groups in combined RCT and LTE data; expressed as incidence rate ratios with 95% CIs and depicted graphically as a forest plot. Exposure is reported in person-years. The relative weighting of each study from a random-effects model is shown. A fixed continuity correction of 0.1 was used for studies with zero events. Heterogeneity between studies was assessed using I² statistics. Further details and references for included studies are provided within Supplementary Table S1. JAKi: Janus kinase inhibitor; TNFi: Tumour Necrosis Factor-alpha inhibitor; RCT: randomised clinical trial; LTE: long-term extension.

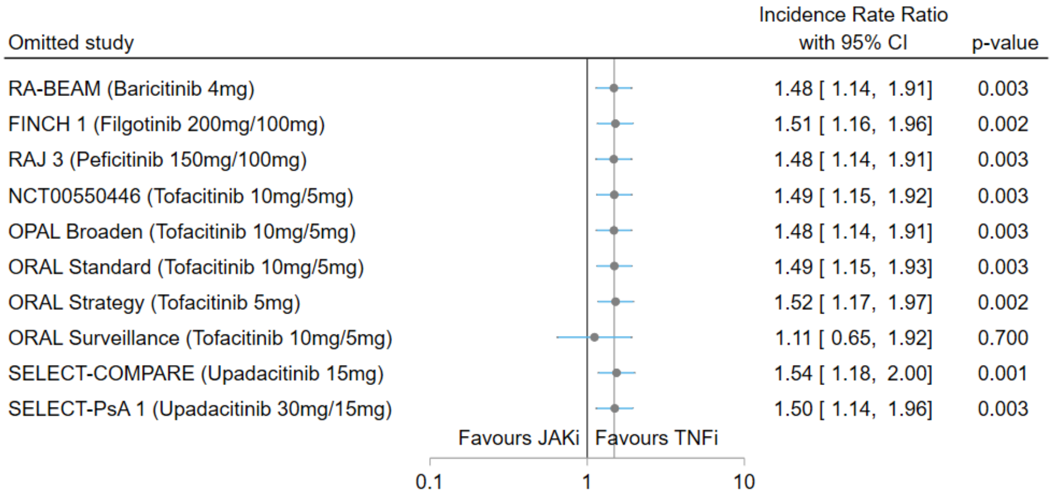

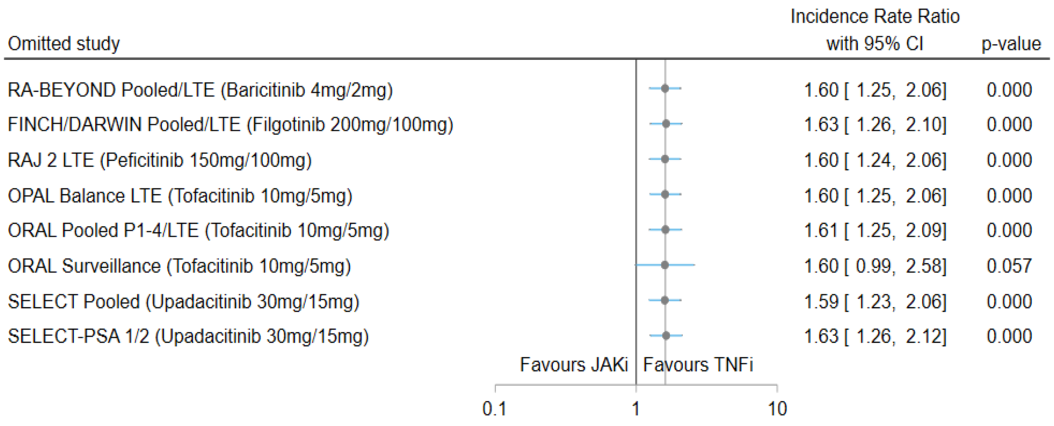

Supplementary Figure S13. Sensitivity analysis demonstrating the influence of individual studies on pairwise meta-analysis results for all malignancy events, comparing JAKi and TNFi groups of eligible RCTs (top panel) and combined RCT and LTE studies (bottom panel). The effect estimates (incidence rate ratio, 95% CIs, p-values) provided on each row correspond to the meta-analysis results when excluding that study. A fixed continuity correction of 0.1 was used for studies with zero events. JAKi: Janus kinase inhibitor; TNFi: Tumour Necrosis Factor-alpha inhibitor.

Study	Treatment Events	Treatment Exposure	Control Events	Control Exposure	Incidence Rate Ratio with 95% CI	Weight (%)
RA-BEYOND Pooled/LTE (Baricitinib 4mg/2mg)	189	14,744	1	161	1.87 [0.29, 12.1]	11.88
FINCH/DARWIN Pooled/LTE (Filgotinib 200mg/100mg)	69	8,085	5	334	0.56 [0.23, 1.37]	41.12
ORAL Pooled P1-4/LTE (Tofacitinib 10mg/5mg)	306	22,875	1	212	2.56 [0.40, 16.6]	11.89
SELECT Pooled (Upadacitinib 30mg/15mg)	66	4,020	4	369	1.47 [0.55, 3.97]	35.12
**Overall**					1.09 [0.56, 2.13]	

Heterogeneity: $\tau^2 = 0.08$, $I^2 = 15.60\%$, $H^2 = 1.18$

Test of $\theta = 0$: $z = 0.25$, $p = 0.80$

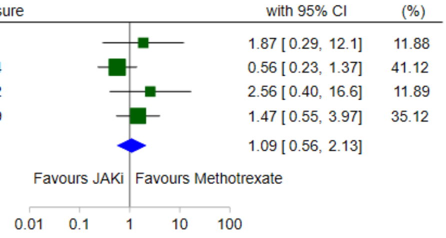

Fixed continuity correction of 0.1 applied to studies with zero events

Supplementary Figure S14. Pairwise meta-analysis of the risk of all malignancies including non-melanomatous skin cancers between JAKi and methotrexate groups in combined RCT and LTE data; expressed as incidence rate ratios with 95% CIs and depicted graphically as a forest plot. Exposure is reported in person-years. The relative weighting of each study from a random-effects model is shown. A fixed continuity correction of 0.1 was used for studies with zero events. Heterogeneity between studies was assessed using I² statistics. Further details and references for included studies are provided within Supplementary Table S1. JAKi: Janus kinase inhibitor; RCT: randomised clinical trial; LTE: long-term extension.

References

1. Kremer J, Li ZG, Hall S, et al. Tofacitinib in combination with nonbiologic disease-modifying antirheumatic drugs in patients with active rheumatoid arthritis: a randomized trial. *Ann Intern Med* 2013;159(4):253-61. doi: 10.7326/0003-4819-159-4-201308200-00006
2. Gladman D, Rigby W, Azevedo VF, et al. Tofacitinib for Psoriatic Arthritis in Patients with an Inadequate Response to TNF Inhibitors. *N Engl J Med* 2017;377(16):1525-36. doi: 10.1056/NEJMoa1615977
3. Mease P, Hall S, FitzGerald O, et al. Tofacitinib or Adalimumab versus Placebo for Psoriatic Arthritis. *N Engl J Med* 2017;377(16):1537-50. doi: 10.1056/NEJMoa1615975
4. Sandborn WJ, Su C, Sands BE, et al. Tofacitinib as Induction and Maintenance Therapy for Ulcerative Colitis. *N Engl J Med* 2017;376(18):1723-36. doi: 10.1056/NEJMoa1606910
5. Deodhar A, Sliwinska-Stanczyk P, Xu H, et al. Tofacitinib for the treatment of ankylosing spondylitis: a phase III, randomised, double-blind, placebo-controlled study. *Annals of the Rheumatic Diseases* 2021;80(8):1004-13. doi: 10.1136/annrheumdis-2020-219601
6. Fleischmann R, Kremer J, Cush J, et al. Placebo-controlled trial of tofacitinib monotherapy in rheumatoid arthritis. *N Engl J Med* 2012;367(6):495-507. doi: 10.1056/NEJMoa1109071
7. van Vollenhoven RF, Fleischmann R, Cohen S, et al. Tofacitinib or Adalimumab versus Placebo in Rheumatoid Arthritis. *New England Journal of Medicine* 2012;367(6):508-19. doi: 10.1056/NEJMoa1112072
8. Lee EB, Fleischmann R, Hall S, et al. Tofacitinib versus Methotrexate in Rheumatoid Arthritis. *New England Journal of Medicine* 2014;370(25):2377-86. doi: 10.1056/NEJMoa1310476
9. Fleischmann R, Mysler E, Hall S, et al. Efficacy and safety of tofacitinib monotherapy, tofacitinib with methotrexate, and adalimumab with methotrexate in patients with rheumatoid arthritis (ORAL Strategy): a phase 3b/4, double-blind, head-to-head, randomised controlled trial. *Lancet* 2017;390(10093):457-68. doi: 10.1016/S0140-6736(17)31618-5 [published Online First: 20170616]
10. Papp KA, Menter MA, Abe M, et al. Tofacitinib, an oral Janus kinase inhibitor, for the treatment of chronic plaque psoriasis: results from two randomized, placebo-controlled, phase III trials. *British Journal of Dermatology* 2015;173(4):949-61. doi: https://doi.org/10.1111/bjd.14018
11. Panés J, Sandborn WJ, Schreiber S, et al. Tofacitinib for induction and maintenance therapy of Crohn's disease: results of two phase IIb randomised placebo-controlled trials. *Gut* 2017;66(6):1049-59. doi: 10.1136/gutjnl-2016-312735
12. Burmester GR, Blanco R, Charles-Schoeman C, et al. Tofacitinib (CP-690,550) in combination with methotrexate in patients with active rheumatoid arthritis with an inadequate response to tumour necrosis factor inhibitors: a randomised phase 3 trial. *Lancet* 2013;381(9865):451-60. doi: 10.1016/S0140-6736(12)61424-X [published Online First: 20130105]
13. Zhang J, Tsai TF, Lee MG, et al. The efficacy and safety of tofacitinib in Asian patients with moderate to severe chronic plaque psoriasis: A Phase 3, randomized, double-blind, placebo-controlled study. *J Dermatol Sci* 2017;88(1):36-45. doi: 10.1016/j.jdermsci.2017.05.004 [published Online First: 20170516]
14. van der Heijde D, Strand V, Tanaka Y, et al. Tofacitinib in Combination With Methotrexate in Patients With Rheumatoid Arthritis: Clinical Efficacy, Radiographic, and Safety Outcomes From a Twenty-Four–Month, Phase III Study. *Arthritis & Rheumatology* 2019;71(6):878-91. doi: https://doi.org/10.1002/art.40803
15. Ytterberg SR, Bhatt DL, Mikuls TR, et al. Cardiovascular and Cancer Risk with Tofacitinib in Rheumatoid Arthritis. *New England Journal of Medicine* 2022;386(4):316-26. doi: 10.1056/NEJMoa2109927
16. Kremer JM, Cohen S, Wilkinson BE, et al. A phase IIb dose-ranging study of the oral JAK inhibitor tofacitinib (CP-690,550) versus placebo in combination with background methotrexate in patients with active rheumatoid arthritis and an inadequate response to methotrexate

alone. *Arthritis Rheum* 2012;64(4):970-81. doi: 10.1002/art.33419 [published Online First: 20111017]

17. van der Heijde D, Deodhar A, Wei JC, et al. Tofacitinib in patients with ankylosing spondylitis: a phase II, 16-week, randomised, placebo-controlled, dose-ranging study. *Annals of the Rheumatic Diseases* 2017;76(8):1340-47. doi: 10.1136/annrheumdis-2016-210322
18. Fleischmann R, Cutolo M, Genovese MC, et al. Phase IIb dose-ranging study of the oral JAK inhibitor tofacitinib (CP-690,550) or adalimumab monotherapy versus placebo in patients with active rheumatoid arthritis with an inadequate response to disease-modifying antirheumatic drugs. *Arthritis Rheum* 2012;64(3):617-29. doi: 10.1002/art.33383
19. Nash P, Coates LC, Fleishaker D, et al. Safety and efficacy of tofacitinib up to 48 months in patients with active psoriatic arthritis: final analysis of the OPAL Balance long-term extension study. *The Lancet Rheumatology* 2021;3(4):e270-e83. doi: 10.1016/S2665-9913(21)00010-2
20. Papp KA, Krueger JG, Feldman SR, et al. Tofacitinib, an oral Janus kinase inhibitor, for the treatment of chronic plaque psoriasis: Long-term efficacy and safety results from 2 randomized phase-III studies and 1 open-label long-term extension study. *J Am Acad Dermatol* 2016;74(5):841-50. doi: 10.1016/j.jaad.2016.01.013 [published Online First: 20160219]
21. Sandborn WJ, D'Haens GR, Sands BE, et al. OP38 Tofacitinib for the treatment of Ulcerative Colitis: An integrated summary of safety data from the global OCTAVE and RIVETING clinical trials. *Journal of Crohn's and Colitis* 2022;16(Supplement_1):i044-i45. doi: 10.1093/ecco-jcc/jjab232.037
22. Cohen SB, Tanaka Y, Mariette X, et al. Long-term safety of tofacitinib up to 9.5 years: a comprehensive integrated analysis of the rheumatoid arthritis clinical development programme. *RMD Open* 2020;6(3) doi: 10.1136/rmdopen-2020-001395 [published Online First: 2020/11/01]
23. Dougados M, van der Heijde D, Chen Y-C, et al. Baricitinib in patients with inadequate response or intolerance to conventional synthetic DMARDs: results from the RA-BUILD study. *Annals of the Rheumatic Diseases* 2017;76(1):88-95. doi: 10.1136/annrheumdis-2016-210094
24. Genovese MC, Kremer J, Zamani O, et al. Baricitinib in Patients with Refractory Rheumatoid Arthritis. *New England Journal of Medicine* 2016;374(13):1243-52. doi: 10.1056/NEJMoa1507247
25. Keystone EC, Taylor PC, Drescher E, et al. Safety and efficacy of baricitinib at 24 weeks in patients with rheumatoid arthritis who have had an inadequate response to methotrexate. *Annals of the Rheumatic Diseases* 2015;74(2):333-40. doi: 10.1136/annrheumdis-2014-206478
26. Taylor PC, Keystone EC, van der Heijde D, et al. Baricitinib versus Placebo or Adalimumab in Rheumatoid Arthritis. *N Engl J Med* 2017;376(7):652-62. doi: 10.1056/NEJMoa1608345
27. Fleischmann R, Schiff M, van der Heijde D, et al. Baricitinib, Methotrexate, or Combination in Patients With Rheumatoid Arthritis and No or Limited Prior Disease-Modifying Antirheumatic Drug Treatment. *Arthritis Rheumatol* 2017;69(3):506-17. doi: 10.1002/art.39953
28. Li Z, Hu J, Bao C, et al. Baricitinib in patients with rheumatoid arthritis with inadequate response to methotrexate: results from a phase 3 study. *Clin Exp Rheumatol* 2020;38(4):732-41. [published Online First: 20200520]
29. Tanaka Y, Emoto K, Cai Z, et al. Efficacy and Safety of Baricitinib in Japanese Patients with Active Rheumatoid Arthritis Receiving Background Methotrexate Therapy: A 12-week, Double-blind, Randomized Placebo-controlled Study. *J Rheumatol* 2016;43(3):504-11. doi: 10.3899/jrheum.150613 [published Online First: 20160201]
30. Simpson EL, Lacour JP, Spelman L, et al. Baricitinib in patients with moderate-to-severe atopic dermatitis and inadequate response to topical corticosteroids: results from two randomized monotherapy phase III trials. *Br J Dermatol* 2020;183(2):242-55. doi: 10.1111/bjd.18898 [published Online First: 20200305]

31. Bieber T, Reich K, Paul C, et al. Efficacy and safety of baricitinib in combination with topical corticosteroids in patients with moderate-to-severe atopic dermatitis with inadequate response, intolerance or contraindication to ciclosporin: results from a randomized, placebo-controlled, phase III clinical trial (BREEZE-AD4)*. *British Journal of Dermatology* 2022;187(3):338-52. doi: https://doi.org/10.1111/bjd.21630
32. Simpson EL, Forman S, Silverberg JI, et al. Baricitinib in patients with moderate-to-severe atopic dermatitis: Results from a randomized monotherapy phase 3 trial in the United States and Canada (BREEZE-AD5). *J Am Acad Dermatol* 2021;85(1):62-70. doi: 10.1016/j.jaad.2021.02.028 [published Online First: 20210216]
33. Reich K, Kabashima K, Peris K, et al. Efficacy and Safety of Baricitinib Combined With Topical Corticosteroids for Treatment of Moderate to Severe Atopic Dermatitis: A Randomized Clinical Trial. *JAMA Dermatology* 2020;156(12):1333-43. doi: 10.1001/jamadermatol.2020.3260
34. Bieber T, Thyssen JP, Reich K, et al. Pooled safety analysis of baricitinib in adult patients with atopic dermatitis from 8 randomized clinical trials. *Journal of the European Academy of Dermatology and Venereology* 2021;35(2):476-85. doi: https://doi.org/10.1111/jdv.16948
35. Taylor PC, Takeuchi T, Burmester GR, et al. Safety of baricitinib for the treatment of rheumatoid arthritis over a median of 4.6 and up to 9.3 years of treatment: final results from long-term extension study and integrated database. *Annals of the Rheumatic Diseases* 2022;81(3):335-43. doi: 10.1136/annrheumdis-2021-221276
36. van der Heijde D, Song IH, Pangan AL, et al. Efficacy and safety of upadacitinib in patients with active ankylosing spondylitis (SELECT-AXIS 1): a multicentre, randomised, double-blind, placebo-controlled, phase 2/3 trial. *Lancet* 2019;394(10214):2108-17. doi: 10.1016/S0140-6736(19)32534-6 [published Online First: 20191112]
37. Fleischmann RM, Genovese MC, Enejosa JV, et al. Safety and effectiveness of upadacitinib or adalimumab plus methotrexate in patients with rheumatoid arthritis over 48 weeks with switch to alternate therapy in patients with insufficient response. *Annals of the Rheumatic Diseases* 2019;78(11):1454-62. doi: 10.1136/annrheumdis-2019-215764
38. Smolen JS, Pangan AL, Emery P, et al. Upadacitinib as monotherapy in patients with active rheumatoid arthritis and inadequate response to methotrexate (SELECT-MONOTHERAPY): a randomised, placebo-controlled, double-blind phase 3 study. *Lancet* 2019;393(10188):2303-11. doi: 10.1016/S0140-6736(19)30419-2 [published Online First: 20190523]
39. van Vollenhoven R, Takeuchi T, Pangan AL, et al. Efficacy and Safety of Upadacitinib Monotherapy in Methotrexate-Naive Patients With Moderately-to-Severely Active Rheumatoid Arthritis (SELECT-EARLY): A Multicenter, Multi-Country, Randomized, Double-Blind, Active Comparator-Controlled Trial. *Arthritis Rheumatol* 2020;72(10):1607-20. doi: 10.1002/art.41384 [published Online First: 20200908]
40. Vollenhoven Rv, Takeuchi T, Pangan A, et al. THU0197 MONOTHERAPY WITH UPADACITINIB IN MTX-NAÏVE PATIENTS WITH RHEUMATOID ARTHRITIS: RESULTS AT 48 WEEKS FROM THE SELECT-EARLY STUDY. *Annals of the Rheumatic Diseases* 2019;78(Suppl 2):376-77. doi: 10.1136/annrheumdis-2019-eular.3350
41. Guttman-Yassky E, Teixeira HD, Simpson EL, et al. Once-daily upadacitinib versus placebo in adolescents and adults with moderate-to-severe atopic dermatitis (Measure Up 1 and Measure Up 2): results from two replicate double-blind, randomised controlled phase 3 trials. *Lancet* 2021;397(10290):2151-68. doi: 10.1016/S0140-6736(21)00588-2 [published Online First: 20210521]
42. Simpson EL, Papp KA, Blauvelt A, et al. Efficacy and Safety of Upadacitinib in Patients With Moderate to Severe Atopic Dermatitis: Analysis of Follow-up Data From the Measure Up 1 and Measure Up 2 Randomized Clinical Trials. *JAMA Dermatology* 2022;158(4):404-13. doi: 10.1001/jamadermatol.2022.0029

43. McInnes IB, Anderson JK, Magrey M, et al. Trial of Upadacitinib and Adalimumab for Psoriatic Arthritis. *New England Journal of Medicine* 2021;384(13):1227-39. doi: 10.1056/NEJMoa2022516
44. McInnes IB, Kato K, Magrey M, et al. Upadacitinib in patients with psoriatic arthritis and an inadequate response to non-biological therapy: 56-week data from the phase 3 SELECT-PsA 1 study. *RMD Open* 2021;7(3):e001838. doi: 10.1136/rmdopen-2021-001838
45. Burmester GR, Winthrop K, Blanco R, et al. Safety Profile of Upadacitinib up to 3 Years in Psoriatic Arthritis: An Integrated Analysis of Two Pivotal Phase 3 Trials. *Rheumatol Ther* 2022;9(2):521-39. doi: 10.1007/s40744-021-00410-z [published Online First: 20211230]
46. Mease PJ, Lertratanakul A, Anderson JK, et al. Upadacitinib for psoriatic arthritis refractory to biologics: SELECT-PsA 2. *Annals of the Rheumatic Diseases* 2021;80(3):312-20. doi: 10.1136/annrheumdis-2020-218870
47. Mease PJ, Lertratanakul A, Papp K, et al. POS0196 UPADACITINIB IN PATIENTS WITH PSORIATIC ARTHRITIS REFRACTORY TO BIOLOGIC DISEASE-MODIFYING ANTIRHEUMATIC DRUGS: 56-WEEK DATA FROM THE PHASE 3 SELECT-PSA 2 STUDY. *Annals of the Rheumatic Diseases* 2021;80(Suppl 1):312-14. doi: 10.1136/annrheumdis-2021-eular.1066
48. Danese S, Vermeire S, Zhou W, et al. Upadacitinib as induction and maintenance therapy for moderately to severely active ulcerative colitis: results from three phase 3, multicentre, double-blind, randomised trials. *Lancet* 2022;399(10341):2113-28. doi: 10.1016/S0140-6736(22)00581-5 [published Online First: 20220526]
49. Silverberg JI, de Bruin-Weller M, Bieber T, et al. Upadacitinib plus topical corticosteroids in atopic dermatitis: Week 52 AD Up study results. *J Allergy Clin Immunol* 2022;149(3):977-87 e14. doi: 10.1016/j.jaci.2021.07.036 [published Online First: 20210814]
50. Burmester GR, Kremer JM, Van den Bosch F, et al. Safety and efficacy of upadacitinib in patients with rheumatoid arthritis and inadequate response to conventional synthetic disease-modifying anti-rheumatic drugs (SELECT-NEXT): a randomised, double-blind, placebo-controlled phase 3 trial. *Lancet* 2018;391(10139):2503-12. doi: 10.1016/S0140-6736(18)31115-2 [published Online First: 20180618]
51. Genovese MC, Fleischmann R, Combe B, et al. Safety and efficacy of upadacitinib in patients with active rheumatoid arthritis refractory to biologic disease-modifying anti-rheumatic drugs (SELECT-BEYOND): a double-blind, randomised controlled phase 3 trial. *Lancet* 2018;391(10139):2513-24. doi: 10.1016/S0140-6736(18)31116-4 [published Online First: 20180618]
52. Zeng X, Zhao D, Radominski SC, et al. Upadacitinib in patients from China, Brazil, and South Korea with rheumatoid arthritis and an inadequate response to conventional therapy. *International Journal of Rheumatic Diseases* 2021;24(12):1530-39. doi: https://doi.org/10.1111/1756-185X.14235
53. Kameda H, Takeuchi T, Yamaoka K, et al. Efficacy and safety of upadacitinib in Japanese patients with rheumatoid arthritis (SELECT-SUNRISE): a placebo-controlled phase IIb/III study. *Rheumatology* 2020;59(11):3303-13. doi: 10.1093/rheumatology/keaa084
54. van der Heijde D, Baraliakos X, Sieper J, et al. Efficacy and safety of upadacitinib for active ankylosing spondylitis refractory to biological therapy: a double-blind, randomised, placebo-controlled phase 3 trial. *Annals of the Rheumatic Diseases* 2022:annrheumdis-2022-222608. doi: 10.1136/ard-2022-222608
55. Guttman-Yassky E, Thaci D, Pangan AL, et al. Upadacitinib in adults with moderate to severe atopic dermatitis: 16-week results from a randomized, placebo-controlled trial. *J Allergy Clin Immunol* 2020;145(3):877-84. doi: 10.1016/j.jaci.2019.11.025 [published Online First: 20191129]
56. Katoh N, Ohya Y, Murota H, et al. A phase 3 randomized, multicenter, double-blind study to evaluate the safety of upadacitinib in combination with topical corticosteroids in adolescent and adult patients with moderate-to-severe atopic dermatitis in Japan (Rising Up): An

interim 24-week analysis. *JAAD International* 2022;6:27-36. doi: https://doi.org/10.1016/j.jdin.2021.11.001
57. Cohen SB, van Vollenhoven RF, Winthrop KL, et al. Safety profile of upadacitinib in rheumatoid arthritis: integrated analysis from the SELECT phase III clinical programme. *Ann Rheum Dis* 2021;80(3):304-11. doi: 10.1136/annrheumdis-2020-218510 [published Online First: 2020/10/30]
58. van der Heijde D, Deodhar A, Maksymowych WP, et al. Upadacitinib in active ankylosing spondylitis: results of the 2-year, double-blind, placebo-controlled SELECT-AXIS 1 study and open-label extension. *RMD Open* 2022;8(2):e002280. doi: 10.1136/rmdopen-2022-002280
59. Zeng X, Zhao D, Radominski S, et al. AB0260 LONG-TERM EFFICACY AND SAFETY OF UPADACITINIB IN PATIENTS FROM CHINA, BRAZIL, AND SOUTH KOREA WITH RHEUMATOID ARTHRITIS AND AN INADEQUATE RESPONSE TO CONVENTIONAL SYNTHETIC DISEASE-MODIFYING ANTIRHEUMATIC DRUGS: RESULTS AT 64 WEEKS. *Annals of the Rheumatic Diseases* 2021;80(Suppl 1):1156-57. doi: 10.1136/annrheumdis-2021-eular.1807
60. D'Haens G, Panés J, Louis E, et al. Upadacitinib Was Efficacious and Well-tolerated Over 30 Months in Patients With Crohn’s Disease in the CELEST Extension Study. *Clinical Gastroenterology and Hepatology* 2022;20(10):2337-46.e3. doi: 10.1016/j.cgh.2021.12.030
61. Kameda H, Takeuchi T, Yamaoka K, et al. Efficacy and safety of upadacitinib over 84 weeks in Japanese patients with rheumatoid arthritis (SELECT-SUNRISE). *Arthritis Res Ther* 2021;23(1):9. doi: 10.1186/s13075-020-02387-6 [published Online First: 20210106]
62. Katoh N, Ohya Y, Murota H, et al. Safety and Efficacy of Upadacitinib for Atopic Dermatitis in Japan: 2-Year Interim Results from the Phase 3 Rising Up Study. *Dermatology and Therapy* 2022 doi: 10.1007/s13555-022-00842-7
63. Genovese MC, Kalunian K, Gottenberg J-E, et al. Effect of Filgotinib vs Placebo on Clinical Response in Patients With Moderate to Severe Rheumatoid Arthritis Refractory to Disease-Modifying Antirheumatic Drug Therapy: The FINCH 2 Randomized Clinical Trial. *JAMA* 2019;322(4):315-25. doi: 10.1001/jama.2019.9055
64. U.S. National Library of Medicine. Study to Evaluate the Efficacy and Safety of Filgotinib in the Treatment of Perianal Fistulizing Crohn's Disease (Divergence2) 2022 [Available from: https://clinicaltrials.gov/ct2/show/NCT03077412 accessed 6 October 2022.
65. Westhovens R, Taylor PC, Alten R, et al. Filgotinib (GLPG0634/GS-6034), an oral JAK1 selective inhibitor, is effective in combination with methotrexate (MTX) in patients with active rheumatoid arthritis and insufficient response to MTX: results from a randomised, dose-finding study (DARWIN 1). *Annals of the Rheumatic Diseases* 2017;76(6):998-1008. doi: 10.1136/annrheumdis-2016-210104
66. Kavanaugh A, Kremer J, Ponce L, et al. Filgotinib (GLPG0634/GS-6034), an oral selective JAK1 inhibitor, is effective as monotherapy in patients with active rheumatoid arthritis: results from a randomised, dose-finding study (DARWIN 2). *Annals of the Rheumatic Diseases* 2017;76(6):1009-19. doi: 10.1136/annrheumdis-2016-210105
67. Feagan BG, Danese S, Loftus EV, Jr., et al. Filgotinib as induction and maintenance therapy for ulcerative colitis (SELECTION): a phase 2b/3 double-blind, randomised, placebo-controlled trial. *Lancet* 2021;397(10292):2372-84. doi: 10.1016/S0140-6736(21)00666-8 [published Online First: 20210603]
68. Westhovens R, Rigby WFC, van der Heijde D, et al. Filgotinib in combination with methotrexate or as monotherapy versus methotrexate monotherapy in patients with active rheumatoid arthritis and limited or no prior exposure to methotrexate: the phase 3, randomised controlled FINCH 3 trial. *Annals of the Rheumatic Diseases* 2021;80(6):727-38. doi: 10.1136/annrheumdis-2020-219213
69. Combe B, Kivitz A, Tanaka Y, et al. Filgotinib versus placebo or adalimumab in patients with rheumatoid arthritis and inadequate response to methotrexate: a phase III randomised

clinical trial. *Annals of the Rheumatic Diseases* 2021;80(7):848-58. doi: 10.1136/annrheumdis-2020-219214

70. Mease P, Coates LC, Helliwell PS, et al. Efficacy and safety of filgotinib, a selective Janus kinase 1 inhibitor, in patients with active psoriatic arthritis (EQUATOR): results from a randomised, placebo-controlled, phase 2 trial. *Lancet* 2018;392(10162):2367-77. doi: 10.1016/S0140-6736(18)32483-8 [published Online First: 20181022]
71. van der Heijde D, Baraliakos X, Gensler LS, et al. Efficacy and safety of filgotinib, a selective Janus kinase 1 inhibitor, in patients with active ankylosing spondylitis (TORTUGA): results from a randomised, placebo-controlled, phase 2 trial. *Lancet* 2018;392(10162):2378-87. doi: 10.1016/S0140-6736(18)32463-2 [published Online First: 20181022]
72. Coates LC, Gladman D, Van den Bosch F, et al. Long-term Outcomes with Filgotinib, an Oral Selective Janus Kinase 1 Inhibitor: 100-week Data from an Open-label Extension (OLE) Study in Patients with Active Psoriatic Arthritis (PsA). *Arthritis Rheumatol* 2020;72 (suppl 10)
73. Winthrop K, Tanaka Y, Takeuchi T, et al. POS0235 INTEGRATED SAFETY ANALYSIS UPDATE FOR FILGOTINIB (FIL) IN PATIENTS (PTS) WITH MODERATELY TO SEVERELY ACTIVE RHEUMATOID ARTHRITIS (RA) RECEIVING TREATMENT OVER A MEDIAN OF 2.2 YEARS (Y). *Annals of the Rheumatic Diseases* 2022;81(Suppl 1):354-55. doi: 10.1136/annrheumdis-2022-eular.1631
74. Schreiber S, Loftus Jr EV, Maaser C, et al. DOP37 Efficacy and safety of filgotinib in patients with Ulcerative Colitis stratified by age: Post hoc analysis of the phase 2b/3 SELECTION and SELECTIONLTE studies. *European Crohn's and Colitis Organisation* 2022
75. Takeuchi T, Tanaka Y, Tanaka S, et al. Efficacy and safety of peficitinib (ASP015K) in patients with rheumatoid arthritis and an inadequate response to methotrexate: results of a phase III randomised, double-blind, placebo-controlled trial (RAJ4) in Japan. *Annals of the Rheumatic Diseases* 2019;78(10):1305-19. doi: 10.1136/annrheumdis-2019-215164
76. Tanaka Y, Takeuchi T, Tanaka S, et al. Efficacy and safety of peficitinib (ASP015K) in patients with rheumatoid arthritis and an inadequate response to conventional DMARDs: a randomised, double-blind, placebo-controlled phase III trial (RAJ3). *Annals of the Rheumatic Diseases* 2019;78(10):1320-32. doi: 10.1136/annrheumdis-2019-215163
77. Takeuchi T, Tanaka Y, Iwasaki M, et al. Efficacy and safety of the oral Janus kinase inhibitor peficitinib (ASP015K) monotherapy in patients with moderate to severe rheumatoid arthritis in Japan: a 12-week, randomised, double-blind, placebo-controlled phase IIb study. *Annals of the Rheumatic Diseases* 2016;75(6):1057-64. doi: 10.1136/annrheumdis-2015-208279
78. Sands BE, Sandborn WJ, Feagan BG, et al. Peficitinib, an Oral Janus Kinase Inhibitor, in Moderate-to-severe Ulcerative Colitis: Results From a Randomised, Phase 2 Study. *Journal of Crohn's and Colitis* 2018;12(10):1158-69. doi: 10.1093/ecco-jcc/jjy085
79. Kivitz AJ, Gutierrez-Urena SR, Poiley J, et al. Peficitinib, a JAK Inhibitor, in the Treatment of Moderate-to-Severe Rheumatoid Arthritis in Patients With an Inadequate Response to Methotrexate. *Arthritis Rheumatol* 2017;69(4):709-19. doi: 10.1002/art.39955
80. Genovese MC, Greenwald M, Codding C, et al. Peficitinib, a JAK Inhibitor, in Combination With Limited Conventional Synthetic Disease-Modifying Antirheumatic Drugs in the Treatment of Moderate-to-Severe Rheumatoid Arthritis. *Arthritis & Rheumatology* 2017;69(5):932-42. doi: https://doi.org/10.1002/art.40054
81. Takeuchi T, Tanaka Y, Tanaka S, et al. Safety and Effectiveness of Peficitinib (ASP015K) in Patients with Rheumatoid Arthritis: Final Results (32 Months of Mean Peficitinib Treatment) From a Long-Term, Open-Label Extension Study in Japan, Korea, and Taiwan. *Rheumatol Ther* 2021;8(1):425-42. doi: 10.1007/s40744-021-00280-5 [published Online First: 20210303]
